# First metagenomic sequencing for the analysis of microbial community populations of adults and pupae of *Melophagus ovinus* in Xinjiang, China

**DOI:** 10.3389/fvets.2024.1462772

**Published:** 2024-12-05

**Authors:** Kaijun Huang, Xing Zhang, Na Xiong, Lu Sun, Xiaoqing Zhao, Kun Zhou, Junyuan Wu

**Affiliations:** ^1^College of Animal Science and Technology, Tarim University, Alar, China; ^2^Engineering Laboratory of Tarim Animal Diseases Diagnosis and Control, Xinjiang Production and Construction Corps, Alar, China; ^3^Key Laboratory of Livestock and Forage Resources Utilization around Tarim, Ministry of Agriculture and Rural Affairs, Alar, China

**Keywords:** *Melophagus ovinus*, pupae, metagenomics sequencing, microbial population, Xinjiang

## Abstract

**Introduction:**

*Melophagus ovinus*, a parasite on the body surface of sheep, directly attacks the host through biting and sucking blood and may also transmit pathogens in the process. There are currently only a few studies on the microbial composition of *M. ovinus*, while there are no such studies on pupae.

**Methods:**

In this study, samples AT-1 to AT-4 each contained four *M. ovinus* individuals, while sample AT-5 comprised four *M. ovinus* pupae, all used for metagenomic sequencing and analysis. *Melophagus ovinus* adults and pupae were collected from four regions in Xinjiang, China. DNA was extracted from the samples, amplified, and sequenced using the Illumina Novaseq 6000 System; finally, the sequencing data were analyzed using molecular biology software.

**Results and discussion:**

From all samples, a total of 32 phyla, comprising 372 genera and 1,037 species, were detected. The highest microbial diversity was observed in Kuqa City (AT-2) and Qira County (AT-4). Pupae exhibited 40 unique microbial genera (AT-5) but did not have the highest microbial diversity. Proteobacteria was the dominant phylum in all samples. The dominant genera included *Bartonella*, *Wolbachia*, *Pseudomonas*, and *Arsenophonus*. This is the first study to report most of the bacteria (e.g., Pseudomonas versuta and *Arsenophonus nasoniae*), fungi (e.g., *Saitoella complicata*), viruses (e.g., Orf virus and Wolbachia phage WO), and protozoa (e.g., *Trypanosoma theileri* and *Babesia bigemina*) in *M. ovinus*. This study has enriched the microbial diversity data of *M. ovinus*, and the pathogens it carries may pose a threat to public health safety and the economy of related industries, necessitating further research to develop effective biological control strategies.

## 1 Introduction

*Melophagus ovinus* is a blood-sucking parasite that parasitizes primarily on the body surface of sheep. This ectoparasite lacks wings and has a surface covered with bristles, robust mouthparts, and three pairs of sharp grappling hooks. *Melophagus ovinus* is a member of the Hippoboscidae (Diptera, Hippoboscoidea) (1); it also parasitizes the body surfaces of numerous animals, including sheep, Tibetan antelope (2), goats (3), rabbits (1), donkeys (4), and so on. The life cycle of this parasite consists of three distinct stages: larva, pupa, and wingless adult. Shortly after birth, female *M. ovinus* mates and produces offspring, giving birth to 12–15 offspring in her lifetime (1). The geographic distribution of *M. ovinus* is very wide and it has been reported in Europe [Slovakia (5), France (6), England (7), Hungary (8), Croatia (9), Czechia (10), Poland (11), and Russia (12)], Asia [Turkey (13) and China (14)], South America [Peru (4)], North America [USA (15)], Oceania [Australia (12) and New Zealand (16)] and Africa [Algeria (17) and Ethiopia (18)].

The parasitization of sheep with large amounts of *M. ovinus* results in two serious consequences. First, by biting and sucking blood, it makes the skin of sheep itchy and painful, leading to skin damage, wool loss, agitation, anemia, and weight loss. More severe cases can be secondary to pathogenic microbial infections and cutaneous myiasis (1). The infestation can lead to a loss in the quality and production from sheep products, such as skin, wool, meat, and milk, adversely affecting the economy of the fur and livestock industries. Massive infections of *M. ovinus* led to a loss of 1.6 million USD by two Ethiopian tanneries over 2 years (19).

More importantly, *M. ovinus* may carry a variety of pathogenic microorganisms that may infect the host in the process of sucking blood, such as viruses [e.g., Bluetongue virus (20), Border disease virus (21) and African swine fever virus (22)], *Acinetobacter* spp. (23) [e.g., *Ac. lwoffii* (24)], *Anaplasma* spp. [e.g., *An. ovis*, *An. phagocytophilum*, and *An. bovis* (25)], *Arsenophonus* spp. (26) [e.g., *Ar. melophagi* (27)], *Bartonella* spp. (11) [e.g., *Ba. schoenbuchensis* (6), *Ba. chomelii* (26), and *Ba. melophagi* (4)], *Borrelia* spp. [e.g., *Bo. garinii*, *Bo. spirochetes* belonging to the *Bo. valaisiana*-related group (28), and *Bo. burgdorferi sensu lato* (11)], *Coxiella* spp. [e.g., *Co. burnetii* (29)], *Rickettsia* spp. (8) [e.g., *Ri. raoultii, Ri. slovaca* (30), *Ri. melophagi* (10), *Ri. massiliae*, and *Candidatus Rickettsia barbariae* (14)], *Theileria* spp. [e.g., *Th. ovis* (2) and *Th. luwenshuni* (31)], *Trypanosoma* spp. (11) [e.g., *Tr. theodori* (1) and *Tr. Melophagium* (32)], *Wolbachia* sp. (23), etc.

Most studies on *M. ovinus* have focused specifically on one to three pathogens. Concurrently, few studies have also detected and identified the types of viruses they carry or analyzed their microbial communities. “Candidatus” is a term used to describe microorganisms that have been discovered solely through molecular biological techniques and have not yet been cultured. It is of significant importance for understanding the functions of ecosystems, advancing biotechnology, and promoting human health. *Candidatus Rickettsia barbariae* has also been detected in ticks and fleas (33–35).

Microorganisms colonize the body surfaces, intestines, hemocoels, and even cells of all insects, which can then serve as vectors for the microorganisms. Their microbial composition and abundance can be altered by a variety of conditions, including insect species, sex, developmental stage, blood-sucking behavior, survival strategies, and geographic location (36). Among these conditions, feeding on the blood reduces the bacterial diversity of the insect gut (37, 38). Insects and the microorganisms they carry may be in mutually beneficial symbiotic, parasitic, and competitive relationships. For example, microorganisms promote insect health, synthesize toxins or modify the insect immune system to protect it from pathogens, parasitize insects, and attack other hosts at opportune times (39). The most important function of microorganisms in symbiotic association with insects is the provision of nutrients, digestion and detoxification (40). For instance, the intestinal commensal bacteria of bees can encode a variety of enzymes, enabling bees to more effectively obtain and utilize carbohydrates from pollen. Experimental evidence also suggests that germ-free *Drosophila* larvae raised in the laboratory exhibit significant growth and developmental delays compared to larvae with a normal microbiota (41). Research by He et al. has confirmed that *Citrobacter* in the gut microbiome of *Drosophila orientacea* can produce 3-hexenyl acetate (3-HA), a compound that attracts female *Drosophila* to oviposit on the same fruit (42). Therefore, certain microbial communities of parasites hold great potential as biocontrol agents for pest management.

The study of the microbial community composition and abundance of *M. ovinus* contributes to scientific knowledge concerning its biology and epidemiology. Metagenomic sequencing involves the high-throughput sequencing of the genomes of microbial communities in a sample, and facilitates the study of microbial population structure. This technique is free from the limitations of microbial isolation and pure culture, and can detect microorganisms in trace numbers, providing an effective tool for studying microbial communities in samples. Currently, only four studies have reported using high-throughput sequencing to analyze the microbial composition and diversity in *M. ovinus*. In 2017, Duan et al. (23) researched the characterization of midgut microbial populations in male and female *M. ovinus*, and in 2020, their team (26) extracted DNA from fully satiated, newly hatched and unfed female *M. ovinus* the midgut and whole body for bacterial community studies. Later in 2021, Litov et al. (12) studied the virome of *M. ovinus* and annotated the full genomes of five novel viruses, and also proved that the Aksy-Durug Melophagus sigma virus can replicate in mammalian cells. In 2022, Liu et al. (22) studied adult *M. ovinus* from three regions of Tibet and discovered for the first time, 23 bacterial genera and multiple DNA viruses. In 2023, Zhang et al. (43) sequenced, assembled, and annotated the full genome of *M. ovinus*. They also discovered that contractions and losses of sensory receptors and vision-associated Rhodopsin genes were significant in *M. ovinus*, and could contribute to the removal of host-sequence contamination during the high-throughput sequencing data processing.

In this study, we employed the Illumina Novaseq 6000 System to metagenomic sequencing and analysis of the microbial composition and abundance in adult and pupae of *M. ovinus* from four regions in Xinjiang, China. This is the first report of the use of a technique has been used to study *M. ovinus* in the area surrounding the Taklamakan Desert, the second most mobile desert in the world, as well as the first study of microbial diversity in pupae.

This study aims to explore and analyze the composition of microbial communities within *M. ovinus* at different life cycle stages from various regions through metagenomic sequencing and analytical techniques. The hypothesis is that these microbial communities may impact the host’s adaptability and transmission capabilities. Additionally, the study will reveal the types of microorganisms that coexist with *M. ovinus* and analyze their roles in the life cycle of *Melophagus ovinus*. Through this research, we expect to provide a scientific basis for the discovery of new pathogens, assess their potential threats to public health safety and the economy of animal-related industries, develop effective strategies for preventing and controlling the spread of vector-borne pathogens, and innovate in pest biological control methods. This will provide valuable data support for scientific research and practical applications in related fields.

## 2 Methods

### 2.1 Sample collection and DNA extraction

**Figure 1:**
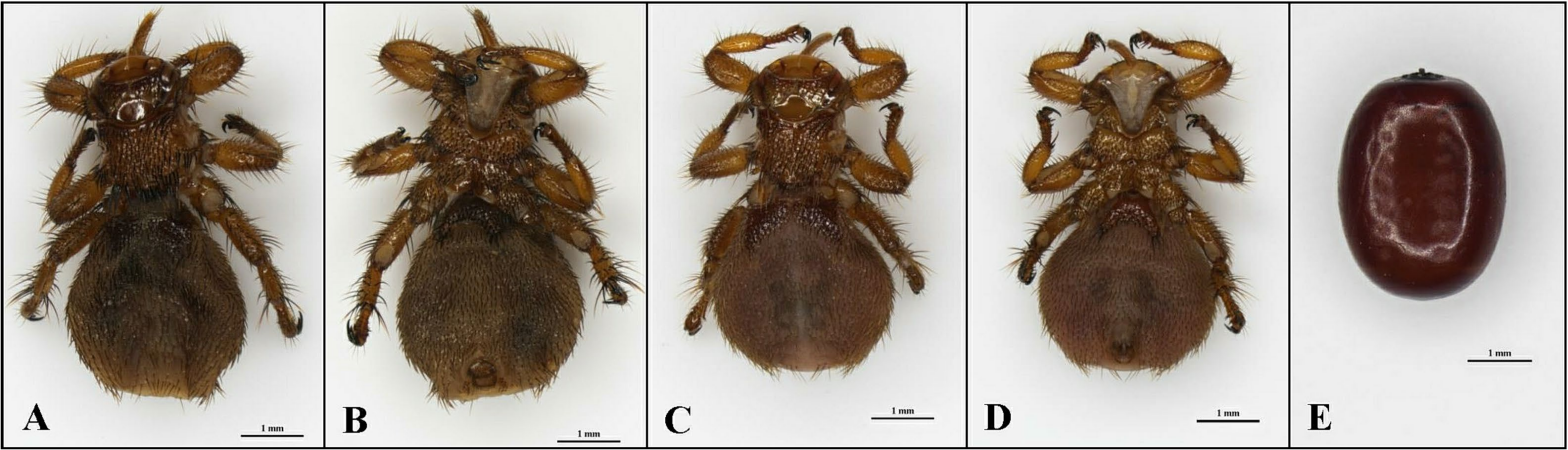
Photomicrographs of adult and pupae of *Melophagus ovinus* (scale bar: 1 mm). **(A)** Back view and **(B)** Ventral view of the female. **(C)** Back view and **(D)** Ventral view of the male. **(E)** Pupae of *M. ovinus.*

*Melophagus ovinus* adults and pupae (Figure 1) were collected from four locations in Xinjiang, China, in 2019 to 2023 (Table 1). All hosts were sheep. *Melophagus ovinus* was collected during the treatment of sheep for ectoparasites. The samples were stored in a −80°C refrigerator until use. *Melophagus ovinus* and its pupae were identified using morphological and molecular biology methods. For better description, the samples will be hence referred to by the abbreviations AT-1, AT-2, AT-3, AT-4, and AT-5 (Table 1).

**Table 1:** Detailed sample information for *Melophagus ovinus* and pupae.

Location	Sample abbreviation	Sample type	Sampling time	Altitude	Longitude and latitude
Urumqi	AT-1	*M. ovinus*	June 2021	580–920 m	43°80′N, 87°60′E
Kuqa City	AT-2	*M. ovinus*	May 2021	930–1,225 m	41°71′N, 82°99′E
Yecheng County	AT-3	*M. ovinus*	March 2019	1,765 m	37°88′N, 77°41′E
Qira County	AT-4	*M. ovinus*	May 2023	2,200–3,200 m	36°26′N, 81°03′E
Qira County	AT-5	Pupae	May 2023	2,200–3,200 m	36°26′N, 81°03′E

This study selected samples based on criteria such as geographical distribution, environmental conditions, developmental stages, and limb integrity. To ensure the comprehensiveness and representativeness of the research results, the samples from the same region consisted of two female and two male adult insects, while the pupal samples were limited in number, allowing for the selection of only four intact individuals for experimentation. The morphological features of *M. ovinus* and pupae are identified using a Leica stereomicroscope M165 C (Solms, Germany) and photomicrographs were taken. All samples were cleaned in ethanol gradient (70, 50, 30, and 10%) for 30 min at 37°C and 180 rpm in a culture oscillator at a constant temperature to remove any remaining debris. The samples were then cleaned three more times in sterile distilled water, and finally dried using a filter paper.

The steps for DNA extraction using the HiPure Soil DNA Kit B (Guangzhou Magen Biotechnology Co., Ltd., China) include: preparing homogenization tubes, bead-beating to lyse the tissue, optional further cell lysis, centrifugation to separate, transferring the supernatant, adding adsorbent and lysis solution, another centrifugation, transferring the supernatant to a new tube, adding buffer SBD, loading onto a DNA column, washing the DNA column, drying the DNA column, and finally eluting DNA with preheated elution buffer and storing it. The extracted DNA was stored at −20°C. Throughout this process, standard protocols are strictly followed to ensure the quality and yield of the DNA. In this study, all experimental operations were conducted within a biosafety cabinet, strictly adhering to aseptic technique protocols. To reduce the potential risk of contamination during the experimental process, both DNA extraction and sequencing steps employed commercial standardized reagents and procedures. Through these measures, we ensured the reliability and reproducibility of the experimental results, providing high-quality DNA templates for subsequent microbial community analysis.

### 2.2 Library preparation and sequencing

Next-generation sequencing libraries were prepared following the manufacturer’s instructions. Genomic DNA (200 μg) was randomly fragmented to an average size of 300–350 bp by Covaris. The fragments were treated using the end-prep enzyme mix for end repair, 5′ phosphorylation, and 3′ adenylated, to add adaptors to both ends. The adaptor-ligated DNA was size-selected by DNA Cleanup beads. Each sample was then amplified by polymerase chain reaction (PCR) for eight cycles using P5 and P7 primers; both primers carry sequences which can anneal with flowcell to perform bridge PCR, while the P7 primer carried a six-base index for multiplexing. The PCR products were cleaned and validated using an Agilent 2100 Bioanalyzer. The qualified libraries were pair-end PE150 sequenced on the Illumina Novaseq 6000 System.

### 2.3 Data analysis

The raw image data of sequencing results were identified (base calling) using the software bcl2fastq (v2.17.1.14), and the quality was preliminarily analyzed to obtain the raw data of sequencing samples (Pass Filter Data), and the data were stored in FASTQ (fq) file format. The quality statistics software cutadapt (v1.9.1) was employed to remove junctions and low-quality sequences from the raw data (Pass Filter Data) (primers and junction sequences were removed; bases with quality values less than 20 at both ends were removed; sequences with N base content greater than 10% were removed; minimum reads length of 75 bp were retained). The samples had contamination from the host, the BWA software (v0.7.12) was used to compare with the host genome, and the reads that might be of host origin were filtered out.

Assembly analysis was performed based on the optimized Clean Data, using MEGAHIT (v1.1.3) software, suitable for large or complex macro-genomic data assembly and was based on the construction of clean de Bruijn plots for low-memory assembly. Different de Bruijin graphs with different K-mer (59, 79, 99, 119, 141) for each sample, were constructed and pre-assembled. Then, the optimal result for that sample was combined with all assembly results as the final assembly result.

The coding gene was predicted using Prodigal (v3.02) software, followed by the integration of gene sequences of all samples and further de-redundant processed by the sequence clustering software MMseq2. The non-redundant gene set unigene sequences were obtained by clustering with 95% identity and 95% coverage by default. The number of reads of unigene in each sample on the alignment was obtained using the alignment software SoapAligner (version 2.21) to compare the clean reads obtained after pre-processing to the constructed non-redundant gene set unigene sequences. Then, based on the number of reads on each unigene pair and the length of the gene, the abundance information of unigene in each sample was estimated.

The microbial composition of the samples was explored by comparing the unigene sequences with the NR database using diamond. The results of species annotation for each sequence were obtained from the information on taxonomic annotation corresponding to each sequence in the NR database. Combining the results of species annotation of genes and the gene abundance tables, information on each species abundance at each taxonomic level (phylum, order, family, genus, species) could be counted for each sample; next, and for the abundance of a given species in a sample, the abundance was summed for genes annotated as that species.

The analysis of *α*-diversity was based on the species-level results, and the α-diversity indices such as Shannon and Chao1 were calculated to represent the species abundance and community diversity by using random sampling of sequences of samples for leveling, and the rank abundance curve were prepared to reflect the species richness as well as evenness.

## 3 Results

### 3.1 General statistics

#### 3.1.1 Sequencing data statistics

After calibration, joints and low-quality sequences of the sequencing results were removed from five samples (AT-1, AT-2, AT-3, AT-4, and AT-5), a total of 86,427,864 optimized data sequences (AT-1, 21,989,176; AT-2, 14,431,232; AT-3, 27,058,326; AT-4, 21,176,652; AT-5, 1,772,478,) were obtained, and the respective length of this optimized data was 12,792,106,261 bp (respectively representing 3,272,729,898 bp, 2,132,276,512 bp, 3,997,349,546 bp, 3,130,724,974 bp, and 259,025,331 bp). The mean length of these optimized sequences was 147.66 bp (respectively representing 148.83 bp, 147.75 bp, 147.73 bp, 147.84 bp, and 146.14 bp).

#### 3.1.2 Alpha-diversity analysis

The AT-2 samples exhibited the highest Ace and Chao1 values, while the AT-4 samples had the highest Shannon and Simpson values. However, as per the guidelines, the Shannon and Simpson values of the AT-2 sample were the most reasonable. The goods coverage value of all the samples was 100%, indicating that the amount of data was sufficient (Table 2).

**Table 2:** Alpha-diversity indices and microbial abundance of the samples.

Sample	Ace	Chao1	Shannon	Simpson	Goods coverage
AT-1	169.276	178	1.541	0.541	1
AT-2	279.023	288.235	1.646	0.483	1
AT-3	207.135	210.667	1.054	0.333	1
AT-4	253.618	276.333	2.106	0.691	1
AT-5	139.065	139	1.615	0.49	1

The greater the Ace index value and the chao1 index value, the greater the number of species in the community. The higher the Shannon index value, the higher the community diversity. The greater the Simpson index value, the lower the community diversity. The larger the goods coverage index value, the lower the probability that the sequence in the sample is not detected.

**Figure 2:**
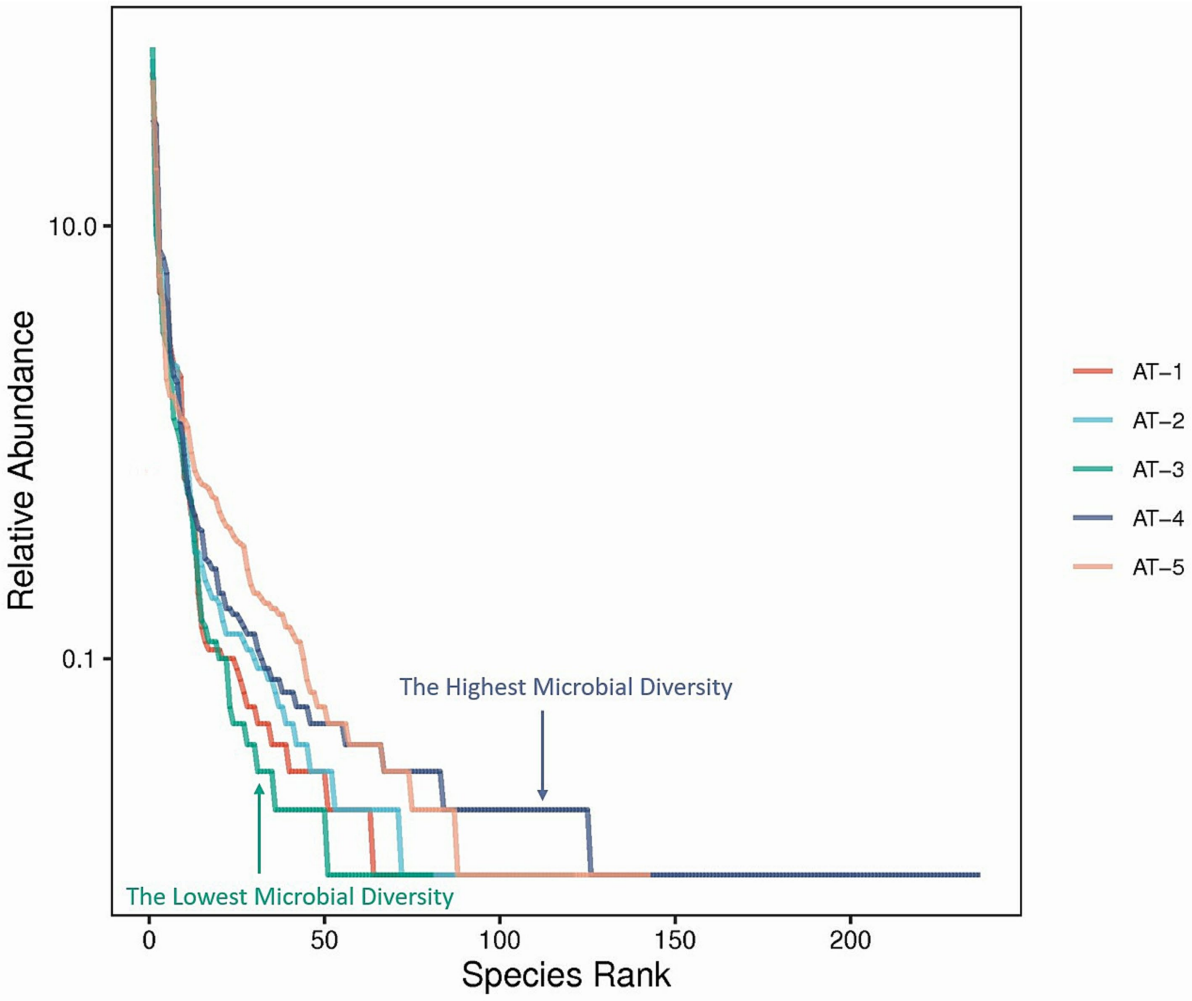
The rank abundance curve. The X-axis presents the rank of the microbial species, and the Y-axis presents the relative abundance of the microbial species. Curves of different colors represent different samples.

The rank abundance curve shows the diversity of each sample. A smoother decline and a longer curve indicate a high diversity of the sample, while a fast and steep decline indicates a high proportion of dominant flora and a low sample diversity. The order of the curve span for the five samples was AT-4 > AT-5 > AT-2 > AT-1 > AT-3, as shown in Figure 2. This result indicates that samples from the sites AT-4 and AT-5 had the highest microbial diversity.

#### 3.1.3 Genus cluster analysis

**Figure 3:**
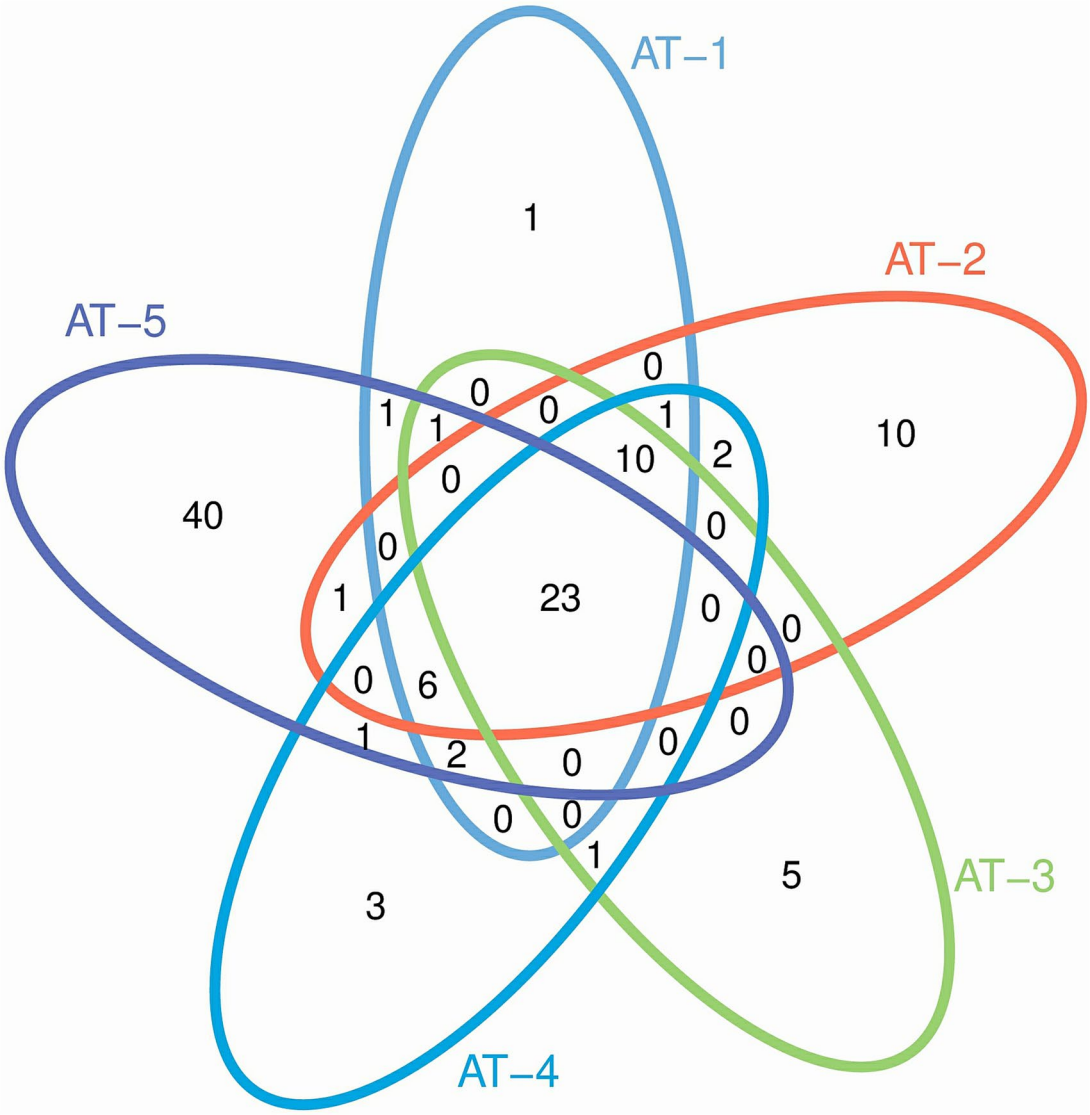
Venn diagrams of microbial abundance in five samples based on Genus.

The Genus cluster analysis is shown as a Venn diagram (Figure 3). The respective numbers of Genus clusters obtained from five samples (AT-1, AT-2, AT-3, AT-4, and AT-5) were 45, 53, 40, 49, and 75. The five samples had 23 highly similar microbial genera, indicating the presence of more of the same microbial populations in the five samples. The AT-5 sample had 40 unique microbial genera, being the highest number of any sample.

### 3.2 Microbial population characteristics

#### 3.2.1 Microbial characteristics at the phylum level

A total of 32 microbial phyla were detected in all samples. Among these, 26, 30, 23, 24, and 20 microbial phyla were detected, respectively, in AT-1, AT-2, AT-3, AT-4, and AT-5. The absolute abundance of these microbial phyla and the community bar plot are presented in Supplementary Table S1 and Supplementary Figure S1A. The dominant phyla were mainly Proteobacteria (respective relative abundance of 94.1, 91.4, 97.3, 92.8, and 93.9%) and Euglenozoa (respective relative abundance of 5.1, 7.3, 1.4, 6.5, and 0.0%). Proteobacteria exhibited a clear advantage across samples. Furthermore, Spirochaetes were present only in AT-1 samples, and Thaumarchaeota, Candidatus Moranbacteria, Verrucomicrobia, and Zoopagomycota were present only in AT-2 samples.

#### 3.2.2 Characteristics of the microbial genera

In all samples, a total of 372 microbial genera were detected, of which 163, 266, 194, 243, and 125 microbial genera were, respectively, detected in AT-1, AT-2, AT-3, AT-4, and AT-5. The absolute abundance of these microbial genera and the community bar plot is presented in Supplementary Table S2 and Supplementary Figure S1B. The relative abundance of *Bartonella* in five samples (AT-1 to AT-5, respectively) was 60.9, 70.2, 80.3, 35.3, and 22.1%. Further, the relative abundance of AT-1, AT-2, and AT-3, was higher than that of AT-4 and AT-5. The relative abundance of *Wolbachia* in AT-5 was 67.8%, being much higher than that in the remaining four samples. A similar situation was noted for the AT-4 sample, with the relative abundance of *Pseudomonas* being 40.8%, also much higher than that in the other four samples. A comparison of relative abundance across samples for *Arsenophonus* (29.0, 13.2, 14.8, 11.1, and 2.8%) and *Trypanosoma* (5.1, 7.2, 1.4, 6.5, and 0.0%) revealed that AT-5 had the lowest relative abundance. The relative abundance of most of the microbial genera, except those mentioned above, was <1%.

#### 3.2.3 Microbial characteristics at the species level

A total of 1,037 microbial species were detected in all samples, of which 302, 622, 464, 639, and 237 microbial species were, respectively, detected in AT-1, AT-2, AT-3, AT-4, and AT-5. The absolute abundance of these microbial species in terms of microbial community bar plot is shown in Supplementary Table S3 and Supplementary Figure S1C. All the samples had a high relative abundance of *Bartonella melophagi* (51.3, 59.1, 66.7, 29.6, and 18.5%), being significantly higher in AT-1, AT-2 and AT-3 than in AT-4 and AT-5. The AT-5 sample had a 47.3% relative abundance of the *Wolbachia* endosymbiont, which was significantly higher than that in the other samples. All the samples had *Arsenophonus nasoniae* (20.0, 9.1, 10.2, 7.7, and 2.0%) but AT-1 had the highest relative abundance. The AT-4 sample had a 31.0% relative abundance of *Pseudomonas versuta* and the rest of the samples was in the range of 0–0.1%. Besides the four bacteria mentioned above, a variety of other bacteria (e.g., *Bartonella bovis* and *Enterobacter cloacae*), archaea (e.g., *Candidatus Nitrosotalea bavarica*), fungi (e.g., *Saitoella complicata*), viruses (e.g., Orf virus and Wolbachia phage WO), and eukaryotes (e.g., *Trypanosoma theileri* and *Babesia bigemina*) were detected in this study, the details of which are presented in Supplementary Table S3.

## 4 Discussion

This study was based on the Illumina Novaseq 6,000 System for metagenomic sequencing. The research examined the microbial population diversity and disparity in four *M. ovinus* samples and one pupae sample obtained from Urumqi (AT-1), Kuqa City (AT-2), Yecheng County (AT-3), and Qira County (AT-4 and AT-5), Xinjiang Uygur Autonomous Region, China. Metagenomic sequencing can detect less abundant microorganisms that cannot be easily culturable, playing an important role in our research. Given the limitations of metagenomic sequencing, we will analyze the results from a more balanced perspective. The results of the study showed that a total of 1,037 species in 32 phyla and 372 genera were detected. After removing the sequences of non-microbial species, 873 microorganisms were detected. Among these, approximately 95 pathogens had varying pathogenicity, representing about 10% of the total. However, most of these pathogens were present in low abundance.

The Alpha-diversity indices and the rank abundance curve showed the richest diversity of microbial populations *M. ovinus* samples from Kuqa City (AT-2) and Qira County (AT-4) contained. Similar to previous studies and as shown by the Venn diagrams, the AT-5 pupae sample, although not the most diverse, had 40 unique genera, consistent with previous results (26). Most of the bacteria, fungi, archaea, spirochetes, chlamydia, viruses, and protozoa in *M. ovinus* and pupae samples are reported for the first time in this study (see Supplementary Table S3 for details). Some microorganisms detected in this study have also been reported previously, such as *Bartonella melophagi*, *Ba. schoenbuchensis, Arsenophonus* endosymbiont, *Wolbachia* endosymbiont, *Escherichia coli*, *Novosphingobium* sp., and so on.

This study compared the findings of previous studies, including those of Duan et al. (23) in 2017, Duan et al. (26) in 2020, and Liu et al. (22) in 2022. For convenience in description, the abbreviations H1, H2, and H3 were used to represent these three studies, respectively. A total of 32 microbial phyla were detected in the present study, while H1, H2, and H3 detected 2, 7, and 5 bacterial phyla, respectively. The dominant bacterial phylum in all studies was Proteobacteria, while Euglenozoa was only found in the present study.

H1, H2, and H3 identified 9, 42, and 29 bacterial genera, respectively, while the present investigation detected 372 genera. These findings align with those of other studies, indicating that *Bartonella*, *Arsenophonus*, and *Wolbachia* were the predominant bacterial genera. Similar to H2, the present study observed that in *M. ovinus* and its pupae the abundance of *Wolbachia* in whole-body samples derived from newly hatched and unfed adult female *M. ovinus* (A.u) was higher than that in whole-body samples obtained from fully engorged adult female *M. ovinus* (A.f), with *Bartonella* showing the opposite trend in abundance. The abundance of *Arsenophonus* in both A.u and A.f found in H2 was around 38%; however, the current study found a lower abundance of *Arsenophonus*, particularly in the pupal samples.

In addition to the influence of geographical factors on the results, the composition of the samples may also be relevant. The samples in H2 were composed exclusively of female *M. ovinus*, whereas the samples in the present study included both male *M. ovinus* and pupal samples of undetermined sex. Individuals with low abundance in the samples can contribute to an overall reduction in abundance.

This study identified a total of 1,037 species, of which 873 were classified as microbes. In contrast, H1, H2, and H3 identified 3, 17, and 7 bacterial species, respectively. The present study observed the same species as H1, including *Pseudomonas aeruginosa* and *Staphylococcus xylosus*, as well as the same *Escherichia coli* as H2; however, the species did not match those detected by H3. The viral metagenomic sequencing used in H3 detected 22 different viruses, the majority of which were phages, a finding that is consistent with the results obtained in the present study.

In summary, the results of the present study have yielded a richer dataset compared with the other three studies, primarily due to the slightly different sequencing techniques employed. By conducting an analysis that spans different times and locations, it is possible to explore variations in the microbial composition of *M. ovinus* across different areas. This also aids in inferring the trends of microbial community evolution and provides a scientific basis for the monitoring and management of *M. ovinus*.

The symbiotic microbial communities of insects are crucial for the host’s adaptability. For instance, the gut microbiota of the *Mormon cricket* is primarily composed of *Lactobacillaceae*, *Enterobacteriaceae*, and *Streptococcaceae*, which influence the host’s adaptability and survival by improving nutrition, enhancing resistance, and regulating social behavior (44); Haloi et al. identified *Bacillus thuringiensis*, *Staphylococcus aureus*, and *Pseudomonas aeruginosa*, and found clear correlations between them and the flacherie disease in *silkworms* (45). The microbial composition of *M. ovinus* is unique compared to other insects, with *Bartonella*, *Wolbachia*, and *Arsenophonus* potentially affecting its health and sex. Future studies should conduct more in-depth research, such as using integrated omics analyses (e.g., proteomics and metabolomics) to systematically analyze the molecular mechanisms of microbe-host interactions.

Symbiotic microorganisms may grow in the gut, body cavity, or cells of insects, potentially causing either positive effects, negative effects, reciprocal effects, or no apparent effect on the host (46). The microbial population composition in insects can vary depending on diet, developmental stage, season, and geography (47). The most abundant microbial genera detected in the study were *Bartonella*, *Arsenophonus*, *Wolbachia*, *Pseudomonas*, and *Trypanosoma*. The microbial species referenced in the subsequent text are all derived from Supplementary Table S3.

Comparing the microbial composition between adult and pupal stages is a meaningful study. Researchers Xue et al. found that *Adelphocoris suturalis* exhibits dynamic changes in gut microbial diversity and composition across different developmental stages, which play a significant role in nutrient absorption and environmental adaptation, and are closely related to the insect’s growth, development, and reproduction (48). This study reveals significant differences in microbial composition between the adult and pupal stages of *M. ovinus*, primarily reflected in the distribution of dominant microbial groups. *Bartonella* dominates from the AT-1 to AT-3 adult stages but shows a marked decrease in relative abundance in the AT-4 and AT-5 pupal samples. This difference may be related to the inconsistency of environmental microbial community composition in high-altitude regions. *Arsenophonus* shows a lower relative abundance, which may suggest that *Wolbachia* influences the sex of *M. ovinus* during the pupal stage, and *Arsenophonus* may affect sex in the adult stage. The higher relative abundance of *Pseudomonas* in the AT-4 samples may be associated with the high abundance of *Pseudomonas* in the adult living environment.

As illustrated in the Venn diagrams (Figure 3), pupae possess a greater number of unique microbial genera compared to adult insects, potentially due to the heightened need for microbial support during their juvenile development for nutrient assimilation and defense against adverse environmental conditions. This analogous situation is also observed in mammals, as exemplified by the Tibetan Sheep (49). As shown in Figure 2, AT-4 and AT-5 have the highest microbial diversity, ranking first and second, respectively. This indicates that the environmental microbiota at the sampling site has a significant impact on the microbial composition of *M. ovinus* and should not be overlooked. Studies have demonstrated that the feeding of different feed components or the inclusion of certain proteases in the diet of ruminants can significantly impact the composition and abundance of rumen microbiota, as well as the metabolites and productive performance (50, 51). *Melophagus ovinus*, being hematophagous arthropods, occasionally bite and ingest blood from various hosts. Consequently, examining the microbial composition and diversity of *M. ovinus* that have fed on different hosts could potentially reveal insights into microbial functionality. Future studies should focus on collecting pupal samples and simultaneously obtaining blood samples from the animal hosts. This will facilitate a deeper investigation into the interactions between microbes, *M. ovinus*, and their animal hosts. Such research not only enhances our understanding of the relationships among these three entities but also provides foundational data for the development of innovative external parasites control strategies and models.

*Bartonella* is a gram-negative bacterium transmitted to humans through blood-sucking arthropod vectors, or contact with contaminated animal feces, or on being scratched by infected animals (52). Some studies have reported *Bartonella* sp. with a high rate of infection in *M. ovinus* samples (6, 11). The last common ancestor of *Bartonella* was a likely amino acid and cofactor self-reliant gut symbiont that recycles nitrogenous waste products from its insect host; these symbionts can adapt to blood-sucking insects, but may not necessarily adapt to mammalian hosts, causing only opportunistic infections (53). We detected *Bartonella melophagi*, *Ba. schoenbuchensis,* and *Ba. bovis*, among others, in our samples. Few studies have successfully isolated and cultured *Bartonella* directly from the bodies of arthropods. The investigation by Kosoy et al. appears to be the only study to have isolated and cultured *Ba. melophagi* from *M. ovinus* suspensions (15). Compared to many previous studies, the current findings on the identification of *Ba. melophagi* are of the highest credibility (22).

*Wolbachia* are considered insect symbionts that on the one hand, help their hosts to resist viruses and insecticides, and also aid in addressing some of the nutritional needs of their hosts (54, 55), on the other hand, enhance their transmission, induce feminization, male-specific killing, and parthenogenesis of insect hosts (56). The relative abundance of *Wolbachia* was as high as 67.8% in AT-5 pupae samples, much higher than that in adult *M. ovinus*. As reported by studied by Duan et al., the abundance of *Wolbachia* in newly hatched and unfed *M. ovinus* was about 29%, also higher than in the adult abundance (26). These values suggest that *Wolbachia* be supposed to have a greater effect on pupae or larvae than on adults.

The genus *Arsenophonus* is a group of symbiont widely present in a several kinds of insects (57). We detected *Arsenophonus nasoniae*, *Ar.* endosymbiont, *Candidatus Arsenophonus lipoptenae*, and *Arsenophonus* sp. ENCA in our study. *Arsenophonus nasoniae* infects people with symptoms of fever and pain (58). Most of these symbionts are vertically transmitted from mother to offspring, while a small percent can also be transmitted horizontally (59). They affect insects and may also make people or animals sick. Therefore, these symbionts need to be researched further.

The bacterium *Pseudomonas* is one of the most ubiquitous and diverse genera across the world, especially in a wide range of environments, objects, and organisms (60). In this study, we detected a variety of *Pseudomonas* sp., with its abundance in the AT-4 adult *M. ovinus* samples being much higher than in other samples. This finding may be related to the suitable altitude and climate of the sample location for its growth. Test results included the opportunistic pathogen *Pseudomonas aeruginosa*, capable of causing serious infections in human respiratory and urinary tracts (61). We also detected a variety of bacterial pathogens, such as *Brucella abortus*, *Acinetobacter baumannii*, *Enterobacter cloacae*, *Salmonella enterica*, *Staphylococcus aureus* and so on, posing a potential threat to human or animal health.

Protozoa have a narrow host range and high specificity, and are thus also used as effective biocontrol agent of pest insects (62). In this study, a total of 45 parasitic protozoa were detected, except for *Trypanosoma*, with a relatively high abundance across samples, while all other protozoa had a relatively low abundance. Until now, no study has reported on the assessment of the interrelationships between *M. ovinus* and protozoa. The protozoa detected in this study are pathogenic, such as *Trypanosoma theileri* is an opportunistic pathogen that can cause mild to severe diseases either independently or in conjunction with other pathogens, sometimes leading to the death of fetuses or newborn calves (63). Following infection with *Babesia bigemina*, the host may exhibit symptoms including fever, anemia, jaundice, loss of appetite, muscle tremors, and death (64).

To date, only five studies have reported the detection of viruses from *M. ovinus* (12, 20–22, 65). In the current study, only the Orf virus and multiple phages were detected. The Orf virus is a DNA virus of genus *Parapoxvirus*, a highly contagious zoonotic disease causing a highly contagious vesiculo ulcerative pustular infection (66). Phages present in *M. ovinus* regulate the bacterial community. The absence of phages may cause an imbalance in the bacterial community in *M. ovinus* and adversely affect its health. In this study only 11 phage species, including Wolbachia phage WO, Escherichia phage L AB-2017, and Staphylococcus phage VB-SauS-SA2, with very low abundance, were detected.

In this study, due to the limited presence of *M. ovinus*, we were unable to collect additional samples from other regions of Xinjiang, including the inability to obtain concurrent pupal samples from the AT-1 to AT-3 sampling sites. Although this is the first study to investigate the symbiotic microbiota of *M. ovinus* in the Xinjiang region using metagenomic sequencing technology, the existing sample size is not sufficient to fully reflect the diversity of microbial species carried by *M. ovinus* across the entire Xinjiang territory. Therefore, future studies will need to collect *M. ovinus* samples from a broader range of areas in Xinjiang to gain a more comprehensive understanding of its microbial symbiosis.

This study identified a variety of congeneric microorganisms (e.g., *Bartonella* spp. and *Trypanosoma* spp.) together with identifying non- relevant species (e.g., *Trichomonas vaginalis* and *Yersinia pestis*). This inaccuracy is likely due to the lower resolution of the metagenomic analysis or DNA barcoding techniques. Therefore, the application of more specific markers is necessary for the identification of these species. For instance, the use of multi-locus sequence typing (MLST) based on the *rpoB*, *gltA*, *ftsZ*, *groEL* genes, and the internal transcribed spacer (ITS) region for the identification of *Bartonella* sp. is warranted (67).

Microorganisms that parasitize insect hosts can positively affect the host, such as providing nutrients or enhancing the fitness of the host while obtaining nutrients for themselves. The microorganisms may also be pathogenic to the insect host, reducing the fitness or causing its death. Multiple studies have demonstrated that antimicrobial resistance is on the rise among various microorganisms, including bacteria and protozoa (68, 69). Consequently, there is an urgent need to develop a bio-inhibitor that is not only safe and economical but also highly effective. Therefore, studying microbial community diversity in *M. ovinus* and determining their roles and functions will make it possible to efficiently investigate a way to manage pests. In this study, multiple pathogens were found in only one or two locations, suggesting that some pathogens may be endemic. During blood-sucking, *M. ovinus* may lead to epidemics of these pathogens in sheep, which in turn may threaten public health safety or the stability of the sheep industry economy.

In this study, a variety of microorganisms were detected, and the data on the microbial diversity of *M. ovinus* in Xinjiang were enriched. However, the data on the microbial diversity of *M. ovinus* in various places is still insignificant to. In conclusion, our study has implications regarding veterinary and public health safety. Based on the pathogen diversity, we need to assess their potential risk of *M. ovinus* to animal husbandry and public health and control its infestations. The relationship, between microorganisms and *M. ovinus* and its transmission and function can be used to develop methods for biological prevention and control of the pests.

## 5 Conclusion

In this study, 32 microbial phyla were detected in *M. ovinus* in Xinjiang, China, with *Proteobacteria* being the dominant phylum. *Bartonella*, *Wolbachia*, *Pseudomonas*, and *Arsenophonus* were the dominant genera among the 372 genera detected. After eliminating non-microbial species, 873 microorganisms, containing nearly 100 pathogens were detected. This is the first study to report most of the bacteria (e.g., *Pseudomonas versuta* and *Arsenophonus nasoniae*), fungi (e.g., *Saitoella complicata*), viruses (e.g., Orf virus and Wolbachia phage WO), and protozoa (e.g., *Trypanosoma theileri* and *Babesia bigemina*) in *M. ovinus*. This study identified the dominant microbial communities in both adult and pupal whole-body samples of *M. ovinus* and revealed differences in microbial diversity and composition across different developmental stages. The findings lay a theoretical foundation for a deeper understanding of the symbiotic relationship between *M. ovinus* and its associated microbes, and provide new insights for the development and innovation of external parasites control strategies in the future.

## Data Availability

The raw tags have been deposited in the Sequence Read Archive (SRA) at the NCBI under the BioProject accession number PRJNA1074431. The individual run files have been assigned accession numbers SRR27908141, SRR27908142, SRR27908143, SRR27908144, and SRR27908145.
