# Supplementary material for: First metagenomic sequencing for the analysis of microbial community populations of adults and pupae of Melophagus ovinus in Xinjiang, China
Source: Front Vet Sci. 2024 Dec 5;11:1462772. doi: 10.3389/fvets.2024.1462772 (PMC11655492; doi:10.3389/fvets.2024.1462772)
Supplement: Supplementary file 1 [file Data_Sheet_1.pdf]

# *Supplementary Material*

## 1 Additional File 1 (Supplementary Tables)

**Table S1.** The absolute abundance of 32 microbial phyla detected in five samples from different parts of China

| Microbial phyla | AT-1 | AT-2 | AT-3 | AT-4 | AT-5 |
|---|---|---|---|---|---|
| Proteobacteria | 1612911 | 1101142 | 2273805 | 1683394 | 87210.5 |
| Euglenozoa | 87700.7 | 87301.6 | 32280.5 | 117316 | 32.2 |
| Arthropoda | 7625.1 | 8341.6 | 4226.5 | 7015 | 4993.3 |
| Actinobacteria | 177.2 | 36.8 | 17906.9 | 102.2 | 55.3 |
| Firmicutes | 2328.2 | 206.8 | 4756 | 2948.7 | 196.5 |
| Streptophyta | 1812 | 1627.2 | 3406.1 | 1227.2 | 40 |
| Chordata | 199.4 | 5375.3 | 44.2 | 2142.9 | 25.1 |
| Unclassified | 237.1 | 343.1 | 478.1 | 142.5 | 65.7 |
| Bacteroidetes | 60.9 | 34.5 | 987.9 | 16.8 | 26.9 |
| Parabasalia | 100 | 100 | 65 | 150 | 170 |
| Acidobacteria | 210.4 | 78.5 | 41.9 | 60.4 | 0 |
| Nematoda | 74.5 | 64.8 | 63 | 91.7 | 47.8 |
| Chlamydiae | 53.1 | 71.9 | 6.2 | 42.5 | 7 |
| Apicomplexa | 86.3 | 23.3 | 10.6 | 38.6 | 6.7 |
| Ascomycota | 3.2 | 68.2 | 4.7 | 23.9 | 9.6 |
| Platyhelminthes | 6.7 | 24.8 | 6 | 22.3 | 0 |
| Chlorophyta | 33 | 8.8 | 8.7 | 5.2 | 0 |
| Nitrospinae | 5 | 7.1 | 7.4 | 10 | 12 |
| Euryarchaeota | 8.8 | 9.1 | 1.7 | 9.9 | 6.4 |
| Mollusca | 4.5 | 11.4 | 3.9 | 10.5 | 3.3 |
| Cyanobacteria | 1.2 | 0 | 3.6 | 11.7 | 4.5 |
| Evosea | 4.2 | 4.2 | 2.7 | 7.1 | 1.7 |
| Mucoromycota | 4 | 7.1 | 3.9 | 3.3 | 0 |
| Haptista | 0 | 1.2 | 0 | 7.2 | 0 |
| Thaumarchaeota | 0 | 7.8 | 0 | 0 | 0 |
| Candidatus Moranbacteria | 0 | 7.7 | 0 | 0 | 0 |
| Candidatus Kaiserbacteria | 4.3 | 2.6 | 0 | 0 | 0 |
| Candidatus Wolfebacteria | 3.3 | 2.9 | 0 | 0 | 0 |
| Planctomycetes | 0 | 3.2 | 0 | 0 | 2.4 |
| Spirochaetes | 5.3 | 0 | 0 | 0 | 0 |
| Verrucomicrobia | 0 | 2.5 | 0 | 0 | 0 |
| Zoopagomycota | 0 | 1.9 | 0 | 0 | 0 |

AT-1: Urumqi, *M. ovinus*; AT-2: Kuqa City, *M. ovinus*; AT-3: Yecheng County, *M. ovinus*; AT-4: Qira County, *M. ovinus*; AT-5: Qira County, Pupae.

**Table S2.** The absolute abundance of 372 microbial genera in the five samples

| Microbial genera | AT-1 | AT-2 | AT-3 | AT-4 | AT-5 |
|---|---|---|---|---|---|
| *Bartonella* | 1043642.7 | 845611.4 | 1877604.3 | 640751.6 | 20518.8 |
| *Arsenophonus* | 497090 | 159320.9 | 345648.3 | 202034.7 | 2627 |
| *Pseudomonas* | 108.2 | 1985.2 | 91.8 | 739483.6 | 20.2 |
| *Trypanosoma* | 87239.8 | 86978.5 | 32137.4 | 117055.2 | 30.8 |
| *Wolbachia* | 51656.1 | 42575.7 | 27123.9 | 58657.1 | 62978 |
| *Pantoea* | 1 | 34035.6 | 8.3 | 25921.9 | 1.2 |
| *Glutamicibacter* | 1 | 0 | 14152.5 | 19.7 | 0 |
| *Lucilia* | 1801.5 | 2274.6 | 1062.3 | 1901.5 | 1175.9 |
| *Beta* | 1800 | 1600 | 3400 | 1200 | 40 |
| *Brucella* | 1800 | 1600 | 3300 | 1100 | 40 |
| *Staphylococcus* | 1092.8 | 64.5 | 4255 | 2091 | 55.2 |
| Unclassified | 1076.6 | 1959.9 | 1456.1 | 2342.2 | 266.1 |
| *Rhizobium* | 1700 | 1400 | 2900 | 1000 | 30 |
| *Escherichia* | 1991.4 | 1033.9 | 2967.8 | 928.2 | 33.9 |
| *Mesorhizobium* | 1500 | 1300 | 2900 | 910 | 40 |
| *Melophagus* | 1940 | 1606 | 817 | 778 | 838 |
| *Xenorhabdus* | 2032 | 743 | 1579 | 934.8 | 19 |
| *Mus* | 0 | 3814 | 0 | 1418.6 | 0 |
| *Enterobacter* | 1701.5 | 819.7 | 1217.3 | 884.9 | 15 |
| *Psychrobacter* | 0 | 45.1 | 1924.2 | 2617 | 0 |
| *Musca* | 796.7 | 1045.1 | 485.8 | 828 | 517.6 |
| *Serratia* | 1300 | 554.9 | 940 | 628.6 | 12 |
| *Erwinia* | 1142.6 | 539.7 | 813.7 | 571.5 | 3 |
| *Trichonephila* | 567.2 | 532.5 | 377 | 864.6 | 633.3 |
| *Moraxella* | 0 | 2872.7 | 7.8 | 17.2 | 0 |
| *Stomoxys* | 594.3 | 756.4 | 365 | 637.2 | 376.9 |
| *Arthrobacter* | 3 | 0 | 2617 | 9.9 | 0 |
| *Providencia* | 1012 | 378.2 | 722.8 | 377.4 | 4.9 |
| *Sodalis* | 810 | 279 | 610 | 357 | 4 |
| *Vollenhovia* | 435.4 | 348.8 | 236.2 | 524.3 | 408.8 |
| *Acinetobacter* | 437 | 819.4 | 402.5 | 201.1 | 1.5 |
| *Lactococcus* | 1001.3 | 56.1 | 52 | 688 | 40 |
| *Drosophila* | 381.9 | 530.8 | 225.8 | 398 | 248.1 |
| *Moellerella* | 610 | 210 | 410 | 260 | 2.2 |
| *Enhydrobacter* | 0 | 1468.2 | 0 | 0 | 0 |
| *Tatumella* | 550 | 216.9 | 420 | 244.2 | 4.3 |
| *Helicobacter* | 643.7 | 29.4 | 14 | 584.5 | 32.2 |
| *Ceratitis* | 272.1 | 355.3 | 176.1 | 293.8 | 183.6 |
| *Yersinia* | 410 | 203.4 | 290 | 347.1 | 1 |
| *Salmonella* | 417.3 | 252 | 329.9 | 193.3 | 2.3 |
| *Photorhabdus* | 410 | 167.2 | 310 | 190 | 1.8 |
| *Gilliamella* | 420 | 143.7 | 260 | 220 | 0 |

| | | | | | |
|---|---|---|---|---|---|
| *Rhagoletis* | 205.2 | 216.7 | 110.5 | 193.2 | 108.6 |
| *Klebsiella* | 172.7 | 152.3 | 137.1 | 348.3 | 3.9 |
| *Chryseobacterium* | 0 | 0 | 782 | 0 | 0 |
| *Bifidobacterium* | 102 | 0 | 553.1 | 45.3 | 52 |
| *Cryptotermes* | 143.3 | 111.2 | 66.9 | 142.6 | 187.2 |
| *Phytomonas* | 280 | 187 | 68.4 | 115 | 0 |
| *Bactrocera* | 118.2 | 167.6 | 93.6 | 135.5 | 76.2 |
| *Trichomonas* | 100 | 100 | 65 | 150 | 170 |
| *Rickettsia* | 124 | 71 | 46.5 | 123 | 151 |
| *Curvibacter* | 59 | 49.3 | 22.3 | 53 | 242.4 |
| *Shigella* | 116.4 | 7.9 | 79.9 | 89 | 6.3 |
| *Rattus* | 0 | 194.8 | 0 | 83.8 | 0 |
| *Zeugodacus* | 54.7 | 77.1 | 38.3 | 65.4 | 32.5 |
| *Exiguobacterium* | 0 | 0 | 264 | 0 | 0 |
| *Obesumbacterium* | 0 | 0 | 0 | 242 | 0 |
| *Leishmania* | 86.8 | 53.1 | 29.2 | 66.1 | 1.4 |
| *Homo* | 0 | 149 | 0 | 64.8 | 0 |
| *Citrobacter* | 3 | 163.2 | 17.1 | 25 | 0 |
| *Flavonifractor* | 45 | 36 | 18 | 48 | 41 |
| *Wasmannia* | 32.3 | 37 | 21.4 | 36 | 58.7 |
| *Pragia* | 0 | 0 | 0 | 170 | 0 |
| *Onchocerca* | 43 | 22 | 15 | 43 | 46 |
| *Paraburkholderia* | 0 | 16 | 0 | 150 | 0 |
| *Chitinophaga* | 0 | 0 | 165 | 0 | 0 |
| *Leptomonas* | 34.6 | 49.9 | 25.1 | 55.2 | 0 |
| *Acidovorax* | 0 | 3.8 | 0 | 150 | 8.9 |
| *Cricetulus* | 0 | 106.5 | 0 | 46.9 | 0 |
| *Avibacterium* | 80.6 | 0 | 24.3 | 9.5 | 36.3 |
| *Methylomonas* | 40.5 | 36.8 | 22.4 | 48.7 | 0 |
| *Pongo* | 0 | 102.1 | 0 | 30.6 | 0 |
| *Enterococcus* | 68.4 | 5.1 | 0 | 45.5 | 3.5 |
| *Chlamydia* | 25.1 | 58.9 | 0 | 35 | 0 |
| *Candidatus Fukatsuia* | 47 | 22 | 6.8 | 16 | 26 |
| *Neotoma* | 0 | 83.7 | 0 | 32.2 | 0 |
| *Caenorhabditis* | 18 | 25 | 37 | 33 | 1.8 |
| *Candidatus Amoebophilus* | 48 | 20 | 11 | 14 | 21 |
| *Candidatus Paracaedibacter* | 43 | 29 | 9.2 | 15 | 14 |
| *Streptomyces* | 61.6 | 14.6 | 16.4 | 12.8 | 0 |
| *Parasteatoda* | 52 | 12 | 9.8 | 9.7 | 18 |
| *Glossina* | 13.9 | 30.3 | 12.9 | 26.7 | 9.5 |
| *Blautia* | 56.9 | 11 | 9.1 | 4.8 | 11.4 |
| *Pteropus* | 0 | 63.5 | 0 | 27 | 0 |
| *Lasius* | 20.8 | 20 | 13.1 | 21.2 | 12.1 |
| *Acromyrmex* | 7.9 | 24 | 24 | 14 | 17 |
| *Bos* | 46.2 | 7.7 | 2.1 | 27.9 | 2.8 |
| *Strigomonas* | 43.2 | 20.7 | 13.6 | 9.2 | 0 |

| | | | | | |
|---|---|---|---|---|---|
| *Ceratina* | 42 | 18 | 6.3 | 6.5 | 11 |
| *Sphaeroforma* | 50 | 9.4 | 9.6 | 8.1 | 0 |
| *Myotis* | 0 | 55.4 | 0 | 16.4 | 0 |
| *Streptococcus* | 4.9 | 0 | 43.3 | 11.7 | 7.1 |
| *Ovis* | 39 | 0 | 2.1 | 23.9 | 0 |
| *Pseudocitrobacter* | 0 | 62 | 0 | 0 | 0 |
| *Neochlamydia* | 28 | 13 | 6.2 | 7.5 | 7 |
| *Eurytemora* | 32 | 14 | 10 | 5.7 | 0 |
| *Ailuropoda* | 0 | 43.1 | 0 | 17.5 | 0 |
| *Babesia* | 42.6 | 0 | 1 | 12.9 | 3 |
| *Capra* | 31.7 | 0 | 1 | 23.2 | 1.4 |
| *Branchiostoma* | 25.5 | 7.7 | 14.4 | 9.4 | 0 |
| *Pan* | 0 | 41.1 | 0 | 15.2 | 0 |
| *Gonium* | 33 | 8.8 | 8.7 | 5.2 | 0 |
| *Bacillus* | 0 | 3.2 | 19.6 | 18.4 | 14.3 |
| *Boleophthalmus* | 13.9 | 11.7 | 6.8 | 10.7 | 11 |
| *Colobus* | 0 | 35 | 0 | 19 | 0 |
| *Saimiri* | 0 | 36 | 0 | 17 | 0 |
| *Saitoella* | 0 | 40 | 0 | 13 | 0 |
| *Bombus* | 14 | 21 | 2.8 | 9.2 | 5.7 |
| *Cryptosporidium* | 31 | 9 | 6.5 | 4.5 | 0 |
| *Angomonas* | 16.3 | 12.4 | 6.8 | 15.3 | 0 |
| *Asbolus* | 8.5 | 13 | 5.4 | 17 | 6 |
| *Epinephelus* | 0 | 36 | 0 | 13 | 0 |
| *Cronobacter* | 0 | 34.5 | 0 | 12.6 | 0 |
| *Comamonas* | 18.3 | 11.3 | 3.7 | 13.7 | 0 |
| *Rhodobacter* | 14 | 13 | 3.7 | 12 | 3.2 |
| *Pectobacterium* | 0 | 22.3 | 0 | 22.9 | 0 |
| *Loxodonta* | 0 | 29.5 | 0 | 15.4 | 0 |
| *Anolis* | 9.6 | 21.6 | 6 | 4.5 | 2.8 |
| *Anopheles* | 8 | 7.9 | 7.8 | 12 | 8.5 |
| *Equus* | 0 | 35.6 | 0 | 8.6 | 0 |
| *Canis* | 0 | 30 | 0 | 14 | 0 |
| *Rhinopithecus* | 0 | 30 | 0 | 14 | 0 |
| *Roseateles* | 0 | 8.8 | 0 | 0 | 35 |
| *Nomascus* | 0 | 34 | 0 | 9.3 | 0 |
| *Athalia* | 5.9 | 15 | 6.1 | 6.8 | 8.1 |
| *Nitrospina* | 5 | 7.1 | 7.4 | 10 | 12 |
| *Eucalyptus* | 5.8 | 14 | 4.6 | 16 | 0 |
| *Laodelphax* | 4.7 | 10 | 4.6 | 14 | 7 |
| *Neisseria* | 6.6 | 15.3 | 0 | 17.8 | 0 |
| *Python* | 0 | 27 | 0 | 12 | 0 |
| *Apis* | 9.2 | 8.7 | 3.4 | 11.1 | 5.7 |
| *Cervus* | 0 | 27 | 0 | 11 | 0 |
| *Stenotrophomonas* | 27.6 | 0 | 10.2 | 0 | 0 |

| | | | | | |
|---|---|---|---|---|---|
| *Mesocricetus* | 0 | 27 | 0 | 10 | 0 |
| *Mycobacteroides* | 0 | 9.2 | 20 | 7.8 | 0 |
| *Blattella* | 7.5 | 11 | 10 | 4.4 | 4 |
| *Buttiauxella* | 0 | 0 | 0 | 36.7 | 0 |
| *Methanosarcina* | 8.8 | 9.1 | 1.7 | 9.9 | 6.4 |
| *Amazona* | 3.3 | 10.5 | 4.7 | 12.2 | 4.6 |
| *Legionella* | 7 | 13 | 2.9 | 7.6 | 4.3 |
| *Patagioenas* | 0 | 23 | 0 | 9.1 | 0 |
| *Vombatus* | 0 | 25.6 | 0 | 6.5 | 0 |
| *Paramormyrops* | 0 | 17.2 | 0 | 14.3 | 0 |
| *Lactobacillus* | 9.8 | 2 | 13 | 4.7 | 1.9 |
| *Trichinella* | 4.3 | 15 | 0 | 12 | 0 |
| *Ooceraea* | 4.6 | 14 | 5.3 | 4.1 | 2.3 |
| *Dipodomys* | 0 | 20.8 | 0 | 9.3 | 0 |
| *Culex* | 3.9 | 8.5 | 2.7 | 5.7 | 8.7 |
| *Eimeria* | 0 | 7.2 | 3.1 | 16 | 1.8 |
| *Agrilus* | 11 | 2.8 | 3.2 | 6.1 | 4.7 |
| *Proteus* | 0 | 13 | 0 | 14.7 | 0 |
| *Raoultella* | 0 | 10 | 0 | 17.1 | 0 |
| *Novimethylophilus* | 0 | 0 | 14 | 13 | 0 |
| *Puma* | 0 | 20 | 0 | 6.9 | 0 |
| *Lelliottia* | 0 | 0 | 0 | 26.8 | 0 |
| *Mandrillus* | 0 | 19 | 0 | 7.5 | 0 |
| *Rahnella* | 0 | 15 | 0 | 10.7 | 0 |
| *Methylocapsa* | 0 | 8.9 | 10 | 5.8 | 1 |
| *Clostridium* | 10.9 | 0 | 0 | 11.9 | 2.8 |
| *Chinchilla* | 0 | 20 | 0 | 5.6 | 0 |
| *Burkholderia* | 11.5 | 3.7 | 1.7 | 7.9 | 0 |
| *Agrococcus* | 0 | 0 | 24.5 | 0 | 0 |
| *Limulus* | 6.4 | 3.8 | 3 | 5.6 | 5.7 |
| *Corvus* | 16.4 | 0 | 0 | 8 | 0 |
| *Xanthomonas* | 23.6 | 0 | 0 | 0 | 0 |
| *Plasmodium* | 11 | 7.1 | 0 | 5.2 | 0 |
| *Yarrowia* | 0 | 23.2 | 0 | 0 | 0 |
| *Sinorhizobium* | 6.3 | 4.7 | 1 | 11 | 0 |
| *Bacteroides* | 6.2 | 1.9 | 7.5 | 1.4 | 5.9 |
| *Macrostomum* | 6.7 | 7.6 | 5 | 3.4 | 0 |
| *Schistosoma* | 0 | 13 | 0 | 9.2 | 0 |
| *Corallococcus* | 14 | 3.8 | 4.1 | 0 | 0 |
| *Kalamiella* | 0 | 15.4 | 0 | 6.3 | 0 |
| *Lipoptena* | 2.8 | 6.8 | 3.8 | 3.7 | 4.6 |
| *Buceros* | 0 | 6.8 | 4.7 | 10 | 0 |
| *Actinobacillus* | 0 | 0 | 3 | 0 | 18.3 |
| *Aedes* | 4.5 | 6.8 | 4.1 | 2.8 | 2.9 |
| *Propithecus* | 0 | 19.5 | 0 | 1 | 0 |
| *Corynebacterium* | 0 | 0 | 19.1 | 1 | 0 |

| | | | | | |
|---|---|---|---|---|---|
| *Haemophilus* | 0 | 11.1 | 0 | 8.9 | 0 |
| *Dictyostelium* | 4.2 | 4.2 | 2.7 | 7.1 | 1.7 |
| *Crassostrea* | 0 | 8 | 1.9 | 9.5 | 0 |
| *Edwardsiella* | 2.6 | 7.6 | 2.9 | 4.5 | 1 |
| *Bicyclus* | 6.3 | 4.8 | 3 | 1.6 | 2.9 |
| *Tupaia* | 0 | 13 | 0 | 5.4 | 0 |
| *Rhizophagus* | 4 | 7.1 | 3.9 | 3.3 | 0 |
| *Hafnia* | 0 | 0 | 0 | 18 | 0 |
| *Rhodanobacter* | 13.4 | 4.5 | 0 | 0 | 0 |
| *Paenibacillus* | 0 | 5.6 | 12.1 | 0 | 0 |
| *Anaerostipes* | 6.4 | 0 | 7.4 | 1 | 2.7 |
| *Neodiprion* | 2 | 6.1 | 2.8 | 6.4 | 0 |
| *Aggregatibacter* | 0 | 0 | 0 | 0 | 17.2 |
| *Paeniglutamicibacter* | 0 | 0 | 16.7 | 0 | 0 |
| *Kluyvera* | 0 | 6.5 | 0 | 10 | 0 |
| *Aspergillus* | 0 | 5 | 1.9 | 9.4 | 0 |
| *Harpegnathos* | 4.8 | 6.6 | 2.2 | 2.5 | 0 |
| *Marinospirillum* | 8 | 5.5 | 2.3 | 0 | 0 |
| *Acipenser* | 1.8 | 4.5 | 0 | 6.8 | 2.5 |
| *Pseudarthrobacter* | 0 | 0 | 11.6 | 3.9 | 0 |
| *Achromobacter* | 0 | 12.4 | 0 | 0 | 2.5 |
| *Trichuris* | 0 | 0 | 11 | 3.7 | 0 |
| *Fonticula* | 5.8 | 1.5 | 1 | 6.3 | 0 |
| *Listeria* | 0 | 1 | 7.3 | 6.1 | 0 |
| *Cimex* | 3.9 | 3.5 | 0 | 4.4 | 2.5 |
| *Elysia* | 4.5 | 3.4 | 2 | 1 | 3.3 |
| *Camelus* | 0 | 8.8 | 0 | 4.9 | 0 |
| *Oryzias* | 0 | 8.3 | 0 | 5.3 | 0 |
| *Schistocephalus* | 0 | 2.6 | 1 | 9.7 | 0 |
| *Leclercia* | 0 | 4.1 | 0 | 9 | 0 |
| *Glaesserella* | 0 | 0 | 0 | 0 | 13 |
| *Gardnerella* | 0 | 7 | 5.7 | 0 | 0 |
| *Pluralibacter* | 0 | 12 | 0 | 0 | 0 |
| *Cyrtomium* | 0 | 4.8 | 0 | 6.5 | 0 |
| *Rubrivivax* | 6.7 | 4.6 | 0 | 0 | 0 |
| *Anaplasma* | 6.1 | 0 | 0 | 5.2 | 0 |
| *Superficieibacter* | 0 | 9.1 | 2 | 0 | 0 |
| *Caballeronia* | 1.4 | 3.6 | 1.5 | 2.9 | 1.6 |
| *Kagunavirus* | 0 | 11 | 0 | 0 | 0 |
| *Phaeobacter* | 0 | 6.9 | 1.3 | 2.7 | 0 |
| *Morganella* | 0 | 3.9 | 4.9 | 2 | 0 |
| *Cyanobacterium* | 1.2 | 0 | 3.6 | 1.5 | 4.5 |
| *Piliocolobus* | 0 | 9 | 0 | 1.7 | 0 |
| *Cardiobacterium* | 0 | 0 | 0 | 10.7 | 0 |
| *Eoetvoesia* | 0 | 1 | 0 | 0 | 9.6 |

| | | | | | |
|---|---|---|---|---|---|
| *Xiphophorus* | 0 | 2.6 | 1.4 | 6.4 | 0 |
| *Trichechus* | 0 | 6.5 | 0 | 3.7 | 0 |
| *Shewanella* | 0 | 9.8 | 0 | 0 | 0 |
| *Galeopterus* | 0 | 9.8 | 0 | 0 | 0 |
| *Subdoligranulum* | 1 | 0 | 8.8 | 0 | 0 |
| *Dickeya* | 0 | 0 | 1 | 8.7 | 0 |
| *Metarhizium* | 0 | 0 | 0 | 0 | 9.6 |
| *Aeromonas* | 0 | 9.5 | 0 | 0 | 0 |
| *Neomonachus* | 0 | 6.5 | 0 | 3 | 0 |
| *Cicer* | 6.2 | 1.8 | 1.5 | 0 | 0 |
| *Dorea* | 6.4 | 1.4 | 1.7 | 0 | 0 |
| *Izhakiella* | 0 | 0 | 0 | 9.4 | 0 |
| *Sextaecvirus* | 0 | 0 | 1.8 | 7.2 | 0 |
| *Rhodoferax* | 0 | 1 | 0 | 0 | 8 |
| *Mixta* | 0 | 0 | 0 | 9 | 0 |
| *Eufriesea* | 4.7 | 1 | 3.3 | 0 | 0 |
| *Callorhinchus* | 0 | 4.9 | 0 | 4 | 0 |
| *Rhodovulum* | 0 | 3.3 | 3.5 | 2 | 0 |
| *Vibrio* | 0 | 0 | 0 | 8.8 | 0 |
| *Felis* | 0 | 5.4 | 0 | 3.3 | 0 |
| *Cedecea* | 0 | 8.5 | 0 | 0 | 0 |
| *Emiliania* | 0 | 1.2 | 0 | 7.2 | 0 |
| *Megachile* | 0 | 2.9 | 1 | 4.4 | 0 |
| *Kocuria* | 0 | 0 | 8.3 | 0 | 0 |
| *Leucobacter* | 0 | 0 | 8.2 | 0 | 0 |
| *Rouxiella* | 0 | 8.1 | 0 | 0 | 0 |
| *Sphingomonas* | 0 | 0 | 0 | 7.9 | 0 |
| *Pedobacter* | 0 | 0 | 7.9 | 0 | 0 |
| *Haematomicrobium* | 0 | 0 | 7.9 | 0 | 0 |
| *Candidatus Nitrosotalea* | 0 | 7.8 | 0 | 0 | 0 |
| *Terrapene* | 0 | 7.8 | 0 | 0 | 0 |
| *Pseudoclavibacter* | 0 | 0 | 7.7 | 0 | 0 |
| *Macrococcus* | 0 | 0 | 6.7 | 1 | 0 |
| *Ruminococcus* | 1.6 | 0 | 6.1 | 0 | 0 |
| *Brenneria* | 0 | 0 | 0 | 7.6 | 0 |
| *Citricoccus* | 0 | 0 | 7.5 | 0 | 0 |
| *Candidimonas* | 0 | 0 | 7.5 | 0 | 0 |
| *Venturia* | 3.2 | 0 | 2.8 | 1.5 | 0 |
| *Riemerella* | 0 | 0 | 7.3 | 0 | 0 |
| *Variovorax* | 0 | 1.8 | 0 | 0 | 5.5 |
| *Spodoptera* | 0 | 2.2 | 0 | 5 | 0 |
| *Ochotona* | 0 | 5.4 | 0 | 1.7 | 0 |
| *Pristionchus* | 4.3 | 2.8 | 0 | 0 | 0 |
| *Parabacteroides* | 0 | 7 | 0 | 0 | 0 |
| *Gallibacterium* | 0 | 5 | 0 | 0 | 1.7 |
| *Limnohabitans* | 0 | 1.4 | 0 | 0 | 5.2 |

| | | | | | |
|---|---|---|---|---|---|
| *Shimwellia* | 0 | 6.5 | 0 | 0 | 0 |
| *Aurantimicrobium* | 0 | 0 | 6.5 | 0 | 0 |
| *Rothia* | 0 | 0 | 6.5 | 0 | 0 |
| *Penaeus* | 0 | 2 | 0 | 4.4 | 0 |
| *Varibaculum* | 0 | 0 | 6.4 | 0 | 0 |
| *Idiomarina* | 4.2 | 0 | 0 | 2.2 | 0 |
| *Cyanistes* | 0 | 6.1 | 0 | 0 | 0 |
| *Nannospalax* | 0 | 4.5 | 0 | 1.6 | 0 |
| *Theropithecus* | 0 | 4.3 | 0 | 1.8 | 0 |
| *Cycloclasticus* | 0 | 0 | 0 | 6.1 | 0 |
| *Mycobacterium* | 0 | 6 | 0 | 0 | 0 |
| *Otolemur* | 0 | 4.4 | 0 | 1.6 | 0 |
| *Coprococcus* | 0 | 1.8 | 2.8 | 0 | 1.3 |
| *Sporosarcina* | 0 | 0 | 5.8 | 0 | 0 |
| *Microcystis* | 0 | 0 | 0 | 5.8 | 0 |
| *Cephalophus* | 4.8 | 0 | 0 | 1 | 0 |
| *Bemisia* | 1 | 1.9 | 0 | 1.9 | 1 |
| *Punavirus* | 0 | 5.7 | 0 | 0 | 0 |
| *Paracoccus* | 0 | 0 | 0 | 0 | 5.6 |
| *Eubacterium* | 3.2 | 0 | 2.3 | 0 | 0 |
| *Pseudoalteromonas* | 0 | 0 | 0 | 5.5 | 0 |
| *Carlito* | 0 | 5.4 | 0 | 0 | 0 |
| *Undibacterium* | 0 | 5.3 | 0 | 0 | 0 |
| *Fusicatenibacter* | 0 | 0 | 4.3 | 1 | 0 |
| *Brachyspira* | 5.3 | 0 | 0 | 0 | 0 |
| *Ralstonia* | 0 | 5.3 | 0 | 0 | 0 |
| *Agathobaculum* | 2.7 | 0 | 1.5 | 0 | 1 |
| *Ruegeria* | 5.2 | 0 | 0 | 0 | 0 |
| *Paroedura* | 4.2 | 0 | 0 | 1 | 0 |
| *Mycolicibacterium* | 5 | 0 | 0 | 0 | 0 |
| *Alligator* | 0 | 3.6 | 0 | 1.4 | 0 |
| *Marinomonas* | 0 | 3.9 | 1 | 0 | 0 |
| *Xenopus* | 0 | 4.8 | 0 | 0 | 0 |
| *Gordonia* | 0 | 0 | 4.8 | 0 | 0 |
| *Nitrosovibrio* | 1 | 0 | 0 | 3.8 | 0 |
| *Meriones* | 0 | 4.7 | 0 | 0 | 0 |
| *Abrus* | 0 | 0 | 0 | 4.7 | 0 |
| *Brachybacterium* | 0 | 0 | 4.6 | 0 | 0 |
| *Roseburia* | 0 | 4.6 | 0 | 0 | 0 |
| *Cajanus* | 0 | 4.6 | 0 | 0 | 0 |
| *Simplicispira* | 3.2 | 1.4 | 0 | 0 | 0 |
| *Clostridioides* | 1 | 0 | 0 | 3.6 | 0 |
| *Alicycliphilus* | 0 | 0 | 0 | 0 | 4.4 |
| *Nostoc* | 0 | 0 | 0 | 4.4 | 0 |
| *Kosakonia* | 0 | 4.4 | 0 | 0 | 0 |

| | | | | | |
|---|---|---|---|---|---|
| *Collinsella* | 0 | 0 | 4.2 | 0 | 0 |
| *Microbacterium* | 0 | 0 | 4.2 | 0 | 0 |
| *Flammeovirga* | 0 | 2.4 | 1.6 | 0 | 0 |
| *Dendroctonus* | 0 | 3.9 | 0 | 0 | 0 |
| *Brugia* | 3.9 | 0 | 0 | 0 | 0 |
| *Shinella* | 0 | 3.9 | 0 | 0 | 0 |
| *Desertihabitans* | 0 | 0 | 3.7 | 0 | 0 |
| *Arcobacter* | 0 | 0 | 0 | 3.6 | 0 |
| *Pasteurella* | 0 | 0 | 1.6 | 1 | 1 |
| *Parapoxvirus* | 3.6 | 0 | 0 | 0 | 0 |
| *Pseudorhodoferax* | 0 | 0 | 0 | 0 | 3.6 |
| *Marinobacter* | 0 | 0 | 3.6 | 0 | 0 |
| *Toxoplasma* | 1.7 | 0 | 0 | 0 | 1.9 |
| *Symbiodinium* | 0 | 1.6 | 0 | 1.9 | 0 |
| *Cavia* | 0 | 3.5 | 0 | 0 | 0 |
| *Novosphingobium* | 0 | 3.5 | 0 | 0 | 0 |
| *Trabulsiella* | 0 | 0 | 3.4 | 0 | 0 |
| *Methyloversatilis* | 0 | 2 | 0 | 0 | 1.4 |
| *Erinaceus* | 0 | 2.3 | 0 | 1 | 0 |
| *Mycetocola* | 0 | 0 | 3.3 | 0 | 0 |
| *Sulfurospirillum* | 0 | 3.2 | 0 | 0 | 0 |
| *Rhodopirellula* | 0 | 3.2 | 0 | 0 | 0 |
| *Ewingella* | 0 | 0 | 3 | 0 | 0 |
| *Octodon* | 0 | 1.9 | 0 | 1 | 0 |
| *Polaromonas* | 0 | 0 | 0 | 0 | 2.7 |
| *Phenylobacterium* | 0 | 2.5 | 0 | 0 | 0 |
| *Alcanivorax* | 0 | 1 | 0 | 1.5 | 0 |
| *Bordetella* | 2.4 | 0 | 0 | 0 | 0 |
| *Aquabacterium* | 0 | 2.3 | 0 | 0 | 0 |
| *Jeotgalicoccus* | 0 | 2.2 | 0 | 0 | 0 |
| *Tyzzerella* | 0 | 0 | 0 | 0 | 2.2 |
| *Gracilibacillus* | 0 | 0 | 0 | 2.2 | 0 |
| *Salpingoeca* | 1 | 1 | 0 | 0 | 0 |
| *Odocoileus* | 2 | 0 | 0 | 0 | 0 |
| *Lipotes* | 0 | 2 | 0 | 0 | 0 |
| *Desmodus* | 0 | 2 | 0 | 0 | 0 |
| *Flavobacterium* | 0 | 0 | 2 | 0 | 0 |
| *Sanguibacter* | 0 | 0 | 2 | 0 | 0 |
| *Candidatus Blochmannia* | 0 | 1.9 | 0 | 0 | 0 |
| *Gulosibacter* | 0 | 0 | 1.9 | 0 | 0 |
| *Smittium* | 0 | 1.9 | 0 | 0 | 0 |
| *Dasypus* | 0 | 1.9 | 0 | 0 | 0 |
| *Methylibium* | 0 | 1.7 | 0 | 0 | 0 |
| *Echinococcus* | 0 | 1.6 | 0 | 0 | 0 |
| *Zhihengliuella* | 0 | 0 | 1.6 | 0 | 0 |
| *Faecalibacterium* | 0 | 1.5 | 0 | 0 | 0 |

| | | | | | |
|---|---|---|---|---|---|
| *Perlucidibaca* | 0 | 1.4 | 0 | 0 | 0 |
| *Peduovirus* | 0 | 0 | 0 | 1.2 | 0 |
| *Corchorus* | 0 | 1 | 0 | 0 | 0 |
| *Francisella* | 0 | 0 | 1 | 0 | 0 |
| *Haemonchus* | 1 | 0 | 0 | 0 | 0 |
| *Parafilimonas* | 1 | 0 | 0 | 0 | 0 |
| *Glycine* | 0 | 1 | 0 | 0 | 0 |
| *Calypte* | 0 | 0 | 1 | 0 | 0 |
| *Oreochromis* | 1 | 0 | 0 | 0 | 0 |
| *Herbaspirillum* | 0 | 0 | 0 | 0 | 1 |
| *Acidocella* | 0 | 1 | 0 | 0 | 0 |

AT-1: Urumqi, *M. ovinus*; AT-2: Kuqa City, *M. ovinus*; AT-3: Yecheng County, *M. ovinus*; AT-4: Qira County, *M. ovinus*; AT-5: Qira County, Pupae.

**Table S3.** The absolute abundance of 1037 microbial species in the above-mentioned five samples

| Microbial species | AT-1 | AT-2 | AT-3 | AT-4 | AT-5 | Is this the first report? |
|---|---|---|---|---|---|---|
| *Bartonella melophagi* | 878967.6 | 712152.8 | 1559884.9 | 536860.4 | 17201.1 | No |
| *Arsenophonus nasoniae* | 342947.2 | 109866.9 | 238752.9 | 139419.9 | 1817.2 | Yes |
| *Pseudomonas versuta* | 44.1 | 1345.8 | 44.1 | 562429.6 | 6.8 | Yes |
| *Bartonella schoenbuchensis* | 76214.7 | 61410.4 | 147921.6 | 48049.1 | 1525 | No |
| Unclassified | 47797.1 | 48109.9 | 75294.6 | 129200.1 | 5529 | Non-microbial |
| *Trypanosoma theileri* | 81847 | 81877.2 | 30081.2 | 110479.1 | 28.7 | Yes |
| *Arsenophonus endosymbiont of Nilaparvata lugens* | 84310 | 26919 | 58523.2 | 34260 | 444.5 | No |
| *Wolbachia endosymbiont of Cimex lectularius* | 34457 | 27745.2 | 17247.7 | 36788.9 | 43942.3 | No |
| *Bartonella* sp. WD12.1 | 36961.2 | 29068.6 | 66933.9 | 22534.1 | 683 | No |
| *Arsenophonus* sp. ENCA | 39287.8 | 12545 | 27183.2 | 15926 | 202.4 | No |
| *Bartonella* sp. WD16.2 | 11367.5 | 9355 | 23285 | 6996.3 | 242.2 | No |
| *Bartonella bovis* | 7891.2 | 6287 | 13555 | 4797.3 | 175.8 | Yes |
| *Arsenophonus endosymbiont of Bemisia tabaci* | 11710 | 3780 | 8130 | 4720 | 62.8 | No |
| *Pantoea vagans* | 0 | 13638.2 | 0 | 11297.2 | 0 | Yes |
| *Arsenophonus endosymbiont of Aleurodicus floccissimus* | 7925 | 2549 | 5439 | 3131.8 | 36.3 | No |
| *Wolbachia pipientis* | 3410.1 | 3285.8 | 2267.9 | 5243.7 | 3918.7 | Yes |
| *Glutamicibacter arilaitensis* | 1 | 0 | 12652.8 | 5.2 | 0 | Yes |
| *Pseudomonas antarctica* | 4.6 | 45.1 | 4.7 | 9250 | 0 | Yes |
| *Trypanosoma grayi* | 2445.3 | 2252 | 877.9 | 2982.3 | 0 | Yes |
| *Wolbachia endosymbiont of Dactylopius coccus* | 1720.7 | 1612.6 | 1078.6 | 2444.8 | 1511 | No |
| *Pseudomonas fragi* | 1.8 | 23.3 | 1.2 | 8339 | 1 | Yes |
| *Lucilia cuprina* | 1801.5 | 2274.6 | 1062.3 | 1901.5 | 1175.9 | Non-microbial |
| *Beta vulgaris* | 1800 | 1600 | 3400 | 1200 | 40 | Non-microbial |
| *Brucella abortus* | 1800 | 1600 | 3300 | 1100 | 40 | Yes |
| *Pseudomonas* sp. KBW05 | 0 | 27.1 | 0 | 7265 | 0 | Yes |
| *Rhizobium* sp. NT-26 | 1700 | 1400 | 2900 | 1000 | 30 | Yes |
| *Escherichia coli* | 1987.3 | 1011.5 | 2851.7 | 924.3 | 31.9 | No |
| *Mesorhizobium loti* | 1500 | 1300 | 2900 | 910 | 40 | Yes |
| *Trypanosoma cruzi* | 1767.5 | 1851.3 | 745.6 | 2279 | 2.1 | Yes |
| *Melophagus ovinus* | 1940 | 1606 | 817 | 778 | 838 | Non-microbial |
| *Wolbachia endosymbiont of Drosophila simulans* | 1076.9 | 908.8 | 688 | 1356.6 | 1343.1 | Yes |
| *Pseudomonas fluorescens* | 0 | 17.9 | 0 | 5129 | 0 | Yes |
| *Mus musculus* | 0 | 3686.6 | 0 | 1382.6 | 0 | Non-microbial |
| *Wolbachia endosymbiont of Armadillidium vulgare* | 1125.5 | 840.3 | 520.8 | 1208.5 | 1202.4 | Yes |
| *Enterobacter cloacae* | 1700 | 598.7 | 1205.4 | 840.3 | 15 | Yes |

| | | | | | | |
|---|---|---|---|---|---|---|
| *Wolbachia endosymbiont of Cylisticus convexus* | 879.5 | 707.5 | 480.2 | 957.5 | 1093.9 | Yes |
| *Pantoea* sp. JKS000250 | 0 | 1590.6 | 1.1 | 2081.8 | 0 | Yes |
| *Musca domestica* | 796.7 | 1045.1 | 485.8 | 828 | 517.6 | Non-microbial |
| *Pseudomonas* sp. PAMC 29040 | 0 | 13.6 | 0 | 3610 | 1 | Yes |
| *Pseudomonas psychrophila* | 0 | 3.5 | 0 | 3551 | 0 | Yes |
| *Serratia marcescens* | 1300 | 529.8 | 940 | 608.1 | 12 | Yes |
| *Staphylococcus equorum* | 0 | 1 | 2413 | 831.4 | 0 | Yes |
| *Pseudomonas* sp. Lz4W | 0 | 8.9 | 0 | 3160 | 0 | Yes |
| *Pantoea agglomerans* | 0 | 2120.3 | 1 | 903.4 | 0 | Yes |
| *Pseudomonas viridiflava* | 0 | 10.3 | 2.1 | 2970 | 0 | Yes |
| *Trichonephila clavipes* | 567.2 | 532.5 | 377 | 864.6 | 633.3 | Non-microbial |
| *Bartonella* sp. AR 15-3 | 640 | 550 | 1300 | 380 | 11 | No |
| *Moraxella osloensis* | 0 | 2812.7 | 0 | 4.2 | 0 | Yes |
| *Bartonella* sp. TT0105 | 0 | 0 | 2300 | 470 | 0 | No |
| *Stomoxys calcitrans* | 594.3 | 756.4 | 365 | 637.2 | 376.9 | Non-microbial |
| *Pseudomonas syringae group genomo*sp. 3 | 0 | 11.5 | 1.5 | 2670 | 0 | Yes |
| *Pseudomonas syringae* | 0 | 39.4 | 1 | 2609 | 0 | Yes |
| *Pseudomonas deceptionensis* | 0 | 12.4 | 2 | 2416 | 0 | Yes |
| *Wolbachia endosymbiont of Laodelphax striatellus* | 510.9 | 432.8 | 286.6 | 608.3 | 585.2 | Yes |
| *Bartonella henselae* | 326 | 588 | 1230 | 274 | 4.2 | Yes |
| *Xenorhabdus bovienii* | 940 | 340 | 760 | 365.6 | 8.5 | Yes |
| *Wolbachia endosymbiont of Brugia malayi* | 618.8 | 410.5 | 253.1 | 476.5 | 597 | Yes |
| *Pantoea* sp. 9140 | 0 | 1057.4 | 0 | 1157.9 | 0 | Yes |
| *Wolbachia endosymbiont of Wuchereria bancrofti* | 454 | 350 | 235.2 | 447.6 | 567.1 | Yes |
| *Wolbachia endosymbiont of Bemisia tabaci* | 513.9 | 358.5 | 206.7 | 415.6 | 508 | Yes |
| *Providencia rustigianii* | 860 | 230 | 580 | 300 | 3 | Yes |
| *Vollenhovia emeryi* | 435.4 | 348.8 | 236.2 | 524.3 | 408.8 | Non-microbial |
| *Lactococcus garvieae* | 1001.3 | 56.1 | 50 | 688 | 40 | Yes |
| *Staphylococcus aureus* | 1036.8 | 38.7 | 24.4 | 642.7 | 31.1 | Yes |
| *Pseudomonas marginalis* | 2.1 | 8.3 | 1.1 | 1720 | 0 | Yes |
| *Trypanosoma rangeli* | 485.5 | 431.9 | 198.1 | 597.5 | 0 | Yes |
| *Sodalis glossinidius* | 660 | 210 | 480 | 290 | 4 | Yes |
| *Pseudomonas poae* | 1 | 1.9 | 0 | 1640 | 0 | Yes |
| *Pseudomonas synxantha* | 0 | 5.8 | 0 | 1516 | 0 | Yes |
| *Moellerella wisconsensis* | 610 | 210 | 410 | 260 | 2.2 | Yes |
| *Wolbachia endosymbiont of Drosophila ananassae* | 330.1 | 242.9 | 148.8 | 355.7 | 371.6 | Yes |
| *Wolbachia endosymbiont of Cadra cautella* | 358.1 | 273.4 | 154.8 | 320.4 | 320.9 | Yes |
| *Pseudomonas* sp. OV657 | 1.2 | 5 | 1 | 1380 | 0 | Yes |

| | | | | | | |
|---|---|---|---|---|---|---|
| *Pantoea* sp. OV426 | 0 | 1208.9 | 0 | 167.4 | 0 | Yes |
| *Helicobacter pylori* | 643.7 | 29.4 | 14 | 584.5 | 32.2 | Yes |
| *Xenorhabdus cabanillasii* | 520 | 190 | 370 | 207.1 | 6.4 | Yes |
| *Xenorhabdus ehlersii* | 500 | 180 | 370 | 240 | 3.1 | Yes |
| *Ceratitis capitata* | 272.1 | 355.3 | 176.1 | 293.8 | 183.6 | Non-microbial |
| *Pseudomonas lundensis* | 0 | 2.5 | 1 | 1209 | 0 | Yes |
| *Candidatus Arsenophonus lipoptenae* | 460 | 170 | 350 | 210 | 3.8 | Yes |
| *Salmonella enterica* | 417.3 | 252 | 327.8 | 193.3 | 2.3 | Yes |
| *Yersinia pestis* | 410 | 151 | 290 | 340 | 1 | Yes |
| *Wolbachia endosymbiont of Diaphorina citri* | 280.3 | 204.2 | 124.3 | 267 | 312.4 | Yes |
| *Pseudomonas* sp. GM48 | 0 | 4.9 | 0 | 1170 | 0 | Yes |
| *Enhydrobacter aerosaccus* | 0 | 1142.3 | 0 | 0 | 0 | Yes |
| *Bartonella rattimassiliensis* | 260 | 220 | 470 | 170 | 8 | Yes |
| *Acinetobacter baumannii* | 437 | 181.3 | 320 | 185.1 | 1.5 | Yes |
| *Wolbachia endosymbiont of Culex quinquefasciatus* | 173.5 | 184.5 | 132.3 | 293 | 340.4 | Yes |
| *Trypanosoma conorhini* | 262.7 | 300.9 | 116.5 | 420 | 0 | Yes |
| *Bartonella* sp. CDC_skunk | 0 | 1 | 860 | 200 | 0 | Yes |
| *Gilliamella apicola* | 420 | 143.7 | 260 | 220 | 0 | Yes |
| *Wolbachia endosymbiont of Ceratosolen solmsi* | 171.5 | 160.9 | 129.7 | 240.8 | 306.2 | Yes |
| *Pseudomonas* sp. 1 R 17 | 0 | 2.8 | 0 | 910 | 0 | Yes |
| *Pseudomonas frederiksbergensis* | 0 | 1.8 | 0 | 870 | 0 | Yes |
| *Rhagoletis zephyria* | 205.2 | 216.7 | 110.5 | 193.2 | 108.6 | Non-microbial |
| *Pseudomonas putida* | 0 | 0 | 0 | 810 | 0 | Yes |
| *Bartonella florencae* | 90 | 193 | 390 | 104 | 5 | Yes |
| *Pseudomonas* sp. NFACC15-1 | 0 | 5 | 0 | 760 | 0 | Yes |
| *Candidatus Erwinia haradaeae* | 300 | 110 | 220 | 120 | 0 | Yes |
| *Wolbachia endosymbiont of Folsomia candida* | 236.9 | 114.6 | 73 | 116.4 | 160.6 | Yes |
| *Pseudomonas taetrolens* | 0 | 1.5 | 0 | 688.6 | 0 | Yes |
| *Pseudomonas lactis* | 0 | 3 | 0 | 681 | 0 | Yes |
| *Psychrobacter* sp. Sarcosine-02u-2 | 0 | 0 | 310.9 | 360.4 | 0 | Yes |
| *Candidatus Erwinia dacicola* | 260 | 101.4 | 190 | 113.3 | 0 | Yes |
| *Cryptotermes secundus* | 143.3 | 111.2 | 66.9 | 142.6 | 187.2 | Non-microbial |
| *Phytomonas* sp. isolate Hart1 | 280 | 187 | 68.4 | 115 | 0 | Yes |
| *Pseudomonas monteilii* | 0 | 0 | 0 | 650 | 0 | Yes |
| *Wolbachia endosymbiont of Glossina morsitans morsitans* | 113 | 110.4 | 68.8 | 189.9 | 165.9 | Yes |
| *Pseudomonas endophytica* | 1.3 | 1 | 0 | 640 | 0 | Yes |
| *Pseudomonas* sp. GLE121 | 0 | 1 | 0 | 640 | 1 | Yes |
| *Pseudomonas arsenicoxydans* | 0 | 4.8 | 0 | 630 | 1 | Yes |
| *Chryseobacterium indoltheticum* | 0 | 0 | 634.6 | 0 | 0 | Yes |
| *Psychrobacter* sp. SHUES1 | 0 | 0 | 288.7 | 327 | 0 | Yes |
| *Pseudomonas* sp. HMWF021 | 0 | 4.8 | 0 | 610 | 0 | Yes |

| | | | | | | |
|---|---|---|---|---|---|---|
| *Wolbachia endosymbiont of Nomada panzeri* | 146.3 | 103.1 | 73.8 | 151.5 | 130.2 | Yes |
| *Pseudomonas chlororaphis* | 0 | 1 | 0 | 584.7 | 0 | Yes |
| *Trichomonas vaginalis* | 100 | 100 | 65 | 150 | 170 | Yes |
| *Wolbachia endosymbiont of Trichogramma pretiosum* | 153 | 94.4 | 54.3 | 143 | 134 | Yes |
| *Wolbachia endosymbiont of Onchocerca volvulus* | 94 | 93 | 63 | 150 | 150 | Yes |
| *Pseudomonas coronafaciens* | 0 | 0 | 0 | 540 | 0 | Yes |
| *Alphaproteobacteria* bacterium | 85.9 | 86 | 56 | 140 | 160 | Yes |
| *Rickettsia endosymbiont of Ixodes pacificus* | 124 | 71 | 46.5 | 123 | 151 | Yes |
| *Bifidobacterium adolescentis* | 66.7 | 0 | 380.9 | 33.4 | 30 | Yes |
| *Arthrobacter* sp. MYb222 | 0 | 0 | 510.8 | 0 | 0 | Yes |
| *Pseudomonas orientalis* | 0 | 0 | 0 | 490 | 0 | Yes |
| *Glutamicibacter* sp. HZAU | 0 | 0 | 471.7 | 2.5 | 0 | Yes |
| *Pseudomonas* sp. URIL14HWK12:I6 | 0 | 1.5 | 0 | 471 | 0 | Yes |
| *Pseudomonas aeruginosa* | 0 | 0 | 0 | 470 | 2.3 | NO |
| *Pseudomonas moorei* | 0 | 0 | 0 | 470 | 0 | Yes |
| *Pseudomonas* sp. AD21 | 0 | 2.5 | 0 | 460 | 0 | Yes |
| *Pseudomonas helleri* | 0 | 0 | 0 | 460 | 0 | Yes |
| *Trypanosoma brucei* | 211 | 103.1 | 48.2 | 93.3 | 0 | Yes |
| *Pseudomonas amygdali* | 0 | 4.5 | 0 | 450 | 0 | Yes |
| *Pseudomonas brassicacearum* | 0 | 1.4 | 0 | 423 | 0 | Yes |
| *Sodalis-like symbiont of Philaenus spumarius* | 150 | 69 | 130 | 67 | 0 | Yes |
| *Trypanosoma vivax* | 125.4 | 98.8 | 42.2 | 145.3 | 0 | Yes |
| *Acidobacteria* bacterium | 210.4 | 78.5 | 41.9 | 60.4 | 0 | Yes |
| *Pseudomonas fuscovaginae* | 0 | 0 | 0 | 390 | 0 | Yes |
| *Klebsiella michiganensis* | 140 | 62.5 | 110 | 73.8 | 1.5 | Yes |
| *Wolbachia endosymbiont of Paratrechina longicornis* | 62 | 69 | 37 | 98 | 120 | Yes |
| *Providencia alcalifaciens* | 150 | 50 | 120 | 64 | 1.9 | Yes |
| *Pseudomonas* sp. CFII64 | 0 | 0 | 1.1 | 380 | 0 | Yes |
| *Wolbachia endosymbiont of Drosophila incompta* | 104.3 | 77.6 | 41 | 102.8 | 48.2 | Yes |
| *Pantoea brenneri* | 0 | 369.7 | 0 | 0 | 0 | Yes |
| *Bactrocera oleae* | 70.5 | 112.8 | 52 | 76.4 | 46.5 | Non-microbial |
| *Pseudomonas* sp. OV546 | 0 | 3.5 | 0 | 350 | 0 | Yes |
| *Wolbachia* sp. wRi_2 | 142.1 | 60 | 31.8 | 55.3 | 63.5 | Yes |
| *Pseudomonas* sp. BGI-2 | 0 | 2.3 | 0 | 350 | 0 | Yes |
| *Klebsiella pneumoniae* | 32.7 | 61.4 | 24.3 | 228.1 | 2.4 | Yes |
| *Wolbachia endosymbiont of Operophtera brumata* | 57.4 | 65.9 | 37.1 | 98 | 90.4 | Yes |
| *Pseudomonas rhodesiae* | 0 | 2.2 | 0 | 330 | 0 | Yes |
| *Pseudomonas grimontii* | 0 | 0 | 0 | 330 | 0 | Yes |

| | | | | | | |
|---|---|---|---|---|---|---|
| *Pseudomonas* sp. 2822-17 | 0 | 2.7 | 0 | 320 | 0 | Yes |
| *Pseudomonas* sp. GM50 | 0 | 2.3 | 0 | 320 | 0 | Yes |
| *Pseudomonas salomonii* | 0 | 0 | 1 | 319 | 0 | Yes |
| *Pseudomonas* sp. ES3-33 | 0 | 0 | 0 | 320 | 0 | Yes |
| *Wolbachia endosymbiont of Drosophila melanogaster* | 71.3 | 65.6 | 46.9 | 73.2 | 57.2 | Yes |
| *Pseudomonas* sp. A46 | 0 | 2.3 | 0 | 310 | 0 | Yes |
| *Pseudomonas* sp. NC02 | 0 | 1.8 | 0 | 310 | 0 | Yes |
| *Pseudomonas* sp. Irchel s3f7 | 0 | 1.4 | 0 | 310 | 0 | Yes |
| *Pseudomonas* sp. DP16D-R1 | 0 | 0 | 0 | 310 | 0 | Yes |
| *Pseudomonas jessenii* | 0 | 0 | 0 | 310 | 0 | Yes |
| *Pseudomonas veronii* | 0 | 1.8 | 0 | 302 | 0 | Yes |
| *Pseudomonas* sp. 655 | 0 | 1.4 | 0 | 302 | 0 | Yes |
| *Pseudomonas* sp. OV081 | 0 | 2.1 | 0 | 300 | 0 | Yes |
| Wolbachia phage WO | 140 | 63 | 21 | 30 | 47 | Yes |
| *Pseudomonas* sp. GM33 | 0 | 1 | 0 | 300 | 0 | Yes |
| *Pseudomonas* sp. 58 R 12 | 0 | 0 | 0 | 300 | 0 | Yes |
| *Pseudomonas savastanoi* | 0 | 0 | 0 | 300 | 0 | Yes |
| *Pseudomonas caspiana* | 0 | 0 | 0 | 300 | 0 | Yes |
| *Pseudomonas* sp. ACN8 | 0 | 1.2 | 0 | 290 | 0 | Yes |
| *Pseudomonas koreensis* | 0 | 0 | 0 | 290 | 0 | Yes |
| *Enhydrobacter* sp. H5 | 0 | 282.2 | 0 | 0 | 0 | Yes |
| *Pseudomonas* sp. IB20 | 0 | 0 | 0 | 280 | 0 | Yes |
| *Rattus norvegicus* | 0 | 194.8 | 0 | 83.8 | 0 | Non-microbial |
| *Pseudomonas lurida* | 0 | 2.1 | 0 | 270 | 0 | Yes |
| *Zeugodacus cucurbitae* | 54.7 | 77.1 | 38.3 | 65.4 | 32.5 | Non-microbial |
| *Wolbachia endosymbiont of Culex molestus* | 62 | 48 | 25.5 | 57.2 | 73.8 | Yes |
| *Pantoea* sp. CFSAN033090 | 0 | 129.1 | 0 | 131 | 0 | Yes |
| *Drosophila kikkawai* | 62 | 61 | 31.9 | 50.2 | 50.5 | Non-microbial |
| *Pseudomonas* sp. Choline-02u-1 | 0 | 1.7 | 0 | 243 | 0 | Yes |
| *Obesumbacterium proteus* | 0 | 0 | 0 | 242 | 0 | Yes |
| *Pseudomonas* sp. 58 R 3 | 0 | 1.8 | 0 | 240 | 0 | Yes |
| *Pseudomonas* sp. Irchel s3h17 | 0 | 1.5 | 0 | 240 | 0 | Yes |
| *Trypanosoma congolense* | 90.2 | 61.3 | 27.7 | 58.7 | 0 | Yes |
| *Drosophila willistoni* | 40.7 | 71.4 | 34 | 52 | 37 | Non-microbial |
| *Pseudomonas* sp. CF161 | 0 | 1.9 | 0 | 230 | 0 | Yes |
| *Pseudomonas* sp. | 0 | 1.5 | 0 | 228 | 0 | Yes |
| *Curvibacter putative symbiont of Hydra magnipapillata* | 59 | 42.5 | 22.3 | 53 | 52 | Yes |
| *Arthrobacter* sp. W1 | 1 | 0 | 226.4 | 0 | 0 | Yes |
| *Pseudomonas vranovensis* | 0 | 3.1 | 0 | 218 | 0 | Yes |
| *Pseudomonas canadensis* | 0 | 0 | 0 | 220 | 0 | Yes |
| *Pantoea* sp. PSNIH6 | 0 | 214.4 | 0 | 1 | 0 | Yes |
| *Homo sapiens* | 0 | 149 | 0 | 64.8 | 0 | Non-microbial |
| *Pseudomonas* sp. Root401 | 0 | 0 | 0 | 212 | 0 | Yes |

| | | | | | | |
|---|---|---|---|---|---|---|
| *Glutamicibacter mysorens* | 0 | 0 | 211.2 | 0 | 0 | Yes |
| *Pseudomonas* sp. o96-267 | 19 | 0 | 0 | 191 | 0 | Yes |
| *Pantoea* sp. MBLJ3 | 0 | 200.6 | 0 | 8.6 | 0 | Yes |
| *Glutamicibacter halophytocola* | 0 | 0 | 207.4 | 0 | 0 | Yes |
| *Pantoea ananatis* | 0 | 100.4 | 0 | 106.8 | 0 | Yes |
| *Shigella sonnei* | 104.4 | 5.4 | 13.3 | 80 | 0 | Yes |
| *Pantoea deleyi* | 0 | 99.2 | 1 | 100.6 | 1.2 | Yes |
| *Pseudomonas* sp. BS3767 | 0 | 0 | 0 | 200 | 0 | Yes |
| *Pseudomonas* sp. A25(2017) | 0 | 0 | 0 | 200 | 0 | Yes |
| *Wolbachia* sp. wRi | 34 | 42.3 | 35.8 | 67.2 | 15.1 | Yes |
| *Pseudomonas weihenstephanensis* | 0 | 2.7 | 0 | 190 | 0 | Yes |
| *Pseudomonas* sp. G5(2012) | 0 | 1.6 | 0 | 190 | 0 | Yes |
| *Arthrobacter* sp. MYb214 | 0 | 0 | 191.5 | 0 | 0 | Yes |
| *Flavonifractor* sp. An112 | 45 | 36 | 18 | 48 | 41 | Yes |
| *Wasmannia auropunctata* | 32.3 | 37 | 21.4 | 36 | 58.7 | Non-microbial |
| *Pseudomonas rhizosphaerae* | 0 | 2.2 | 0 | 180 | 0 | Yes |
| *Pseudomonas* sp. 28 E 9 | 0 | 0 | 0 | 180 | 0 | Yes |
| *Pseudomonas* sp. TMW 2.1634 | 0 | 0 | 0 | 180 | 0 | Yes |
| *Pseudomonas brenneri* | 0 | 0 | 0 | 180 | 0 | Yes |
| *Pseudomonas cuatrocienegasensis* | 0 | 0 | 0 | 180 | 0 | Yes |
| *Psychrobacter cryohalolentis* | 0 | 1 | 75.9 | 99.3 | 0 | Yes |
| *Drosophila hydei* | 43 | 41 | 18.5 | 37 | 33 | Non-microbial |
| *Pseudomonas* sp. 9.1(2019) | 0 | 1.2 | 0 | 169 | 0 | Yes |
| *Pseudomonas extremaustralis* | 0 | 0 | 0 | 170 | 0 | Yes |
| *Pseudomonas* sp. 2588-5 | 0 | 0 | 0 | 170 | 0 | Yes |
| *Pseudomonas psychrotolerans* | 0 | 0 | 0 | 170 | 0 | Yes |
| *Pseudomonas* sp. GR 6-02 | 0 | 0 | 0 | 170 | 0 | Yes |
| *Pseudomonas* sp. FW300-N1A1 | 0 | 0 | 0 | 170 | 0 | Yes |
| *Pseudomonas protegens* | 0 | 0 | 0 | 170 | 0 | Yes |
| *Pseudomonas* sp. TAD18 | 0 | 0 | 0 | 169.5 | 0 | Yes |
| *Onchocerca ochengi* | 42 | 22 | 15 | 43 | 46 | Yes |
| *Chitinophaga* sp. K20C18050901 | 0 | 0 | 165 | 0 | 0 | Yes |
| *Pseudomonas* sp. MYb115 | 0 | 3.9 | 0 | 160 | 0 | Yes |
| *Wolbachia endosymbiont of Nomada ferruginata* | 24.9 | 21 | 23.7 | 54 | 38.9 | Yes |
| *Pseudomonas azotoformans* | 0 | 0 | 0 | 162 | 0 | Yes |
| *Pseudomonas batumici* | 0 | 1.1 | 0 | 160 | 0 | Yes |
| *Pseudomonas abietaniphila* | 0 | 1.1 | 0 | 160 | 0 | Yes |
| *Pseudomonas* sp. ICMP 564 | 0 | 0 | 0 | 160 | 0 | Yes |
| *Pseudomonas* sp. MF4836 | 0 | 0 | 0 | 160 | 0 | Yes |
| *Pseudomonas knackmussii* | 0 | 0 | 0 | 160 | 0 | Yes |
| *Pseudomonas* sp. GM78 | 0 | 0 | 0 | 160 | 0 | Yes |
| *Pseudomonas* sp. Eur1 9.41 | 0 | 0 | 0 | 160 | 0 | Yes |
| *Pseudomonas kilonensis* | 0 | 0 | 0 | 160 | 0 | Yes |
| *Pseudomonas* sp. S06B 330 | 0 | 0 | 0 | 160 | 0 | Yes |

| | | | | | | |
|---|---|---|---|---|---|---|
| *Pseudomonas* sp. R9.37 | 0 | 0 | 0 | 160 | 0 | Yes |
| *Psychrobacter* sp. Sarcosine-3u-12 | 0 | 0 | 73.9 | 82.6 | 0 | Yes |
| *Cricetulus griseus* | 0 | 106.5 | 0 | 46.9 | 0 | Non-microbial |
| *Psychrobacter* sp. PAMC 21119 | 0 | 0 | 25.3 | 127.9 | 0 | Yes |
| *Paraburkholderia tuberum* | 0 | 3 | 0 | 150 | 0 | Yes |
| *Pseudomonas libanensis* | 0 | 1.6 | 0 | 150 | 0 | Yes |
| *Pseudomonas* sp. GM79 | 0 | 1.6 | 0 | 150 | 0 | Yes |
| *Pseudomonas* sp. Larv2_ips | 0 | 1.4 | 0 | 150 | 0 | Yes |
| *Pseudomonas* sp. ACM7 | 0 | 1 | 0 | 150 | 0 | Yes |
| *Pseudomonas* sp. 443 | 0 | 0 | 0 | 150 | 0 | Yes |
| *Pseudomonas* sp. R4-39-08 | 0 | 0 | 0 | 150 | 0 | Yes |
| *Pseudomonas* sp. FW305-53 | 0 | 0 | 0 | 150 | 0 | Yes |
| *Pseudomonas saxonica* | 0 | 0 | 0 | 150 | 0 | Yes |
| *Acidovorax* sp. HMWF029 | 0 | 0 | 0 | 150 | 0 | Yes |
| *Leptomonas pyrrhocoris* | 30 | 46.1 | 21.4 | 51.9 | 0 | Yes |
| *Bactrocera latifrons* | 27 | 37.6 | 26.6 | 32.7 | 22.9 | Non-microbial |
| *Pseudomonas* sp. PE-S1G-1 | 0 | 1.9 | 0 | 140 | 0 | Yes |
| *Arthrobacter* sp. S41 | 0 | 0 | 140 | 0 | 0 | Yes |
| *Pseudomonas* sp. Irchel s3b5 | 0 | 0 | 0 | 140 | 0 | Yes |
| *Pseudomonas* sp. LH1G9 | 0 | 0 | 0 | 140 | 0 | Yes |
| *Pseudomonas* sp. UW4 | 0 | 0 | 0 | 140 | 0 | Yes |
| *Pseudomonas* sp. WP001 | 0 | 0 | 0 | 140 | 0 | Yes |
| *Leishmania braziliensis* | 54.2 | 32 | 21.2 | 31.8 | 0 | Yes |
| *Pantoea conspicua* | 0 | 85.2 | 0 | 53.7 | 0 | Yes |
| *Psychrobacter* sp. DAB_AL32B | 0 | 0 | 83.3 | 55.4 | 0 | Yes |
| *Drosophila busckii* | 36.8 | 45.2 | 16.5 | 28.3 | 8.6 | Non-microbial |
| *Acinetobacter lwoffii* | 0 | 126.8 | 7.5 | 0 | 0 | Yes |
| *Pseudomonas* sp. MWU12-2312b | 0 | 4 | 0 | 130 | 0 | Yes |
| *Pseudomonas* sp. URHB0015 | 0 | 2.7 | 0 | 130 | 0 | Yes |
| *Pongo abelii* | 0 | 102.1 | 0 | 30.6 | 0 | Non-microbial |
| *Pseudomonas* sp. Irchel s3b2 | 0 | 0 | 0 | 130 | 1.2 | Yes |
| *Pseudomonas* sp. MT-1 | 0 | 0 | 0 | 130 | 0 | Yes |
| *Glutamicibacter nicotianae* | 0 | 0 | 128.6 | 0 | 0 | Yes |
| *Psychrobacter* sp. P2G3 | 0 | 8.9 | 44.4 | 72.4 | 0 | Yes |
| *Glutamicibacter* sp. 0426 | 0 | 0 | 125.2 | 0 | 0 | Yes |
| *Bifidobacterium pseudocatenulatum* | 18.9 | 0 | 90.2 | 2.4 | 12.8 | Yes |
| *Psychrobacter arcticus* | 0 | 0 | 67 | 56.8 | 0 | Yes |
| *Pseudomonas stutzeri* | 3.1 | 0 | 0 | 120 | 0 | Yes |
| *Pseudomonas* sp. P1.8 | 0 | 3.1 | 0 | 120 | 0 | Yes |
| *Pseudomonas laurylsulfatiphila* | 0 | 1.2 | 0 | 120 | 0 | Yes |
| *Curvibacter* sp. PD_MW3 | 0 | 5.4 | 0 | 0 | 114.7 | Yes |
| *Staphylococcus pettenkoferi* | 56 | 19 | 9.1 | 13 | 23 | Yes |
| *Pseudomonas* sp. Ag1 | 0 | 0 | 0 | 120 | 0 | Yes |
| *Pseudomonas* sp. ERMR1:02 | 0 | 0 | 0 | 120 | 0 | Yes |
| *Pseudomonas* sp. R2-37-08W | 0 | 0 | 0 | 120 | 0 | Yes |
| *Pseudomonas* sp. LBUM920 | 0 | 0 | 0 | 120 | 0 | Yes |

| | | | | | | |
|---|---|---|---|---|---|---|
| *Pseudomonas* sp. Leaf48 | 0 | 0 | 0 | 120 | 0 | Yes |
| *Pseudomonas* sp. FH1 | 0 | 0 | 0 | 120 | 0 | Yes |
| *Glutamicibacter* sp. BW77 | 0 | 0 | 107.5 | 12 | 0 | Yes |
| *Staphylococcus cohnii* | 0 | 2.5 | 94.3 | 21.5 | 0 | Yes |
| *Candidatus Fukatsuia symbiotica* | 47 | 22 | 6.8 | 16 | 26 | Yes |
| *Neotoma lepida* | 0 | 83.7 | 0 | 32.2 | 0 | Non-microbial |
| *Mus caroli* | 0 | 88.2 | 0 | 26.7 | 0 | Non-microbial |
| *Caenorhabditis remanei* | 18 | 25 | 37 | 33 | 1.8 | Yes |
| *Pantoea* sp. aB | 0 | 99.4 | 0 | 15 | 0 | Yes |
| *Candidatus Amoebophilus asiaticus* | 48 | 20 | 11 | 14 | 21 | Yes |
| *Pantoea* sp. ARC607 | 0 | 91.5 | 0 | 21.8 | 0 | Yes |
| *Wolbachia endosymbiont of Drosophila subpulchrella* | 52 | 18 | 8.1 | 13 | 22 | Yes |
| *Enterobacter hormaechei* | 0 | 96 | 7.7 | 8.5 | 0 | Yes |
| *Pseudomonas* sp. CMAA1215 | 0 | 1.1 | 0 | 110 | 0 | Yes |
| *Pseudomonas cedrina* | 0 | 1 | 0 | 110 | 0 | Yes |
| *Candidatus Paracaedibacter acanthamoebae* | 43 | 29 | 9.2 | 15 | 14 | Yes |
| *Pseudomonas* sp. GL93 | 0 | 0 | 0 | 110 | 0 | Yes |
| *Pseudomonas* sp. B26(2017) | 0 | 0 | 0 | 110 | 0 | Yes |
| *Pseudomonas* sp. Irchel 3H3 | 0 | 0 | 0 | 110 | 0 | Yes |
| *Drosophila virilis* | 17 | 53.9 | 5.4 | 22.2 | 10.2 | Non-microbial |
| *Arthrobacter* sp. EpRS66 | 0 | 0 | 106.4 | 1 | 0 | Yes |
| *Drosophila arizonae* | 23 | 38.1 | 9.9 | 16.3 | 19.2 | Yes |
| *Drosophila eugracilis* | 26.6 | 30.3 | 11.3 | 25.7 | 11.4 | Non-microbial |
| *Psychrobacter cibarius* | 0 | 0 | 57.4 | 47.8 | 0 | Yes |
| *Citrobacter freundii* | 0 | 86.2 | 0 | 18.8 | 0 | Yes |
| *Psychrobacter* sp. G | 0 | 0 | 48.3 | 55.6 | 0 | Yes |
| *Parasteatoda tepidariorum* | 52 | 12 | 9.8 | 9.7 | 18 | Non-microbial |
| *Pseudomonas cichorii* | 1 | 0 | 0 | 100 | 0 | Yes |
| *Psychrobacter* sp. DAB_AL43B | 0 | 0 | 44.3 | 55.8 | 0 | Yes |
| *Pseudomonas asplenii* | 0 | 0 | 0 | 100 | 0 | Yes |
| *Wolbachia endosymbiont of Callosobruchus chinensis* | 13.9 | 17.8 | 16.8 | 36 | 10.5 | Yes |
| *Avibacterium paragallinarum* | 56.6 | 0 | 24.3 | 2.3 | 10.8 | Yes |
| *Exiguobacterium undae* | 0 | 0 | 93.4 | 0 | 0 | Yes |
| *Glossina morsitans* | 13.9 | 30.3 | 12.9 | 26.7 | 9.5 | Non-microbial |
| *Pantoea rwandensis* | 0 | 92.5 | 0 | 0 | 0 | Yes |
| *Chlamydia trachomatis* | 0 | 58.9 | 0 | 30.9 | 0 | Yes |
| *Pantoea anthophila* | 0 | 49.3 | 0 | 39 | 0 | Yes |
| *Pantoea* sp. 3_1284 | 0 | 88 | 0 | 0 | 0 | Yes |
| *Lasius niger* | 20.8 | 20 | 13.1 | 21.2 | 12.1 | Non-microbial |
| *Acromyrmex echinatior* | 7.9 | 24 | 24 | 14 | 17 | Non-microbial |
| *Ceratina calcarata* | 42 | 18 | 6.3 | 6.5 | 11 | Non-microbial |
| *Drosophila obscura* | 17 | 21.2 | 14.5 | 18.1 | 11.7 | Non-microbial |

| | | | | | | |
|---|---|---|---|---|---|---|
| *Psychrobacter aquaticus* | 0 | 0 | 27.2 | 53.9 | 0 | Yes |
| *Bos mutus* | 45.2 | 3.8 | 2.1 | 26.9 | 2.8 | Non-microbial |
| *Pantoea* sp. 3.5.1 | 0 | 77.3 | 0 | 2.9 | 0 | Yes |
| *Arthrobacter* sp. MYb213 | 0 | 0 | 72.6 | 6.9 | 0 | Yes |
| *Drosophila rhopaloa* | 17.6 | 14.9 | 9.4 | 28.8 | 8.6 | Non-microbial |
| *synthetic construct* | 0 | 58 | 0 | 20.7 | 0 | Non-microbial |
| *Pantoea dispersa* | 0 | 48.5 | 0 | 28.8 | 0 | Yes |
| *Sphaeroforma arctica* | 50 | 9.4 | 9.6 | 8.1 | 0 | Non-microbial |
| *Psychrobacter* sp. JCM 18903 | 0 | 1 | 24 | 52 | 0 | Yes |
| *Wolbachia endosymbiont of Nomada flava* | 14.8 | 14.9 | 10.5 | 28 | 8.4 | Yes |
| *Enterococcus faecalis* | 41 | 5.1 | 0 | 25.9 | 2.5 | Yes |
| *Strigomonas culicis* | 43.2 | 16.5 | 9.7 | 4.9 | 0 | Yes |
| Wolbachia endosymbiont wVitB of Nasonia vitripennis phage WOVitB | 31 | 15 | 7.5 | 8.8 | 12 | Yes |
| *Drosophila biarmipes* | 15 | 19.5 | 14.4 | 15.2 | 9.2 | Non-microbial |
| *Pseudomonas prosekii* | 0 | 0 | 0 | 71 | 0 | Yes |
| *Streptomyces* sp. or3 | 43 | 11 | 9.5 | 6.3 | 0 | Yes |
| *[Ruminococcus] gnavus* | 53.2 | 7.8 | 1.2 | 1.2 | 6.1 | Yes |
| *Pteropus alecto* | 0 | 47 | 0 | 21.6 | 0 | Non-microbial |
| *Psychrobacter immobilis* | 0 | 0 | 41.2 | 25.6 | 0 | Yes |
| *Pseudomonas gingeri* | 0 | 1 | 0 | 65 | 0 | Yes |
| *Ovis aries* | 39 | 0 | 2.1 | 23.9 | 0 | Non-microbial |
| *Drosophila melanogaster* | 9.4 | 20.9 | 7.4 | 20.1 | 5.6 | Non-microbial |
| *Xenorhabdus innexi* | 0 | 0 | 0 | 62.5 | 0 | Yes |
| *Arthrobacter* sp. NIO-1057 | 0 | 0 | 62 | 0 | 0 | Yes |
| *Psychrobacter* sp. P11F6 | 0 | 0 | 37.7 | 24.3 | 0 | Yes |
| *Pseudocitrobacter* sp. RIT 415 | 0 | 62 | 0 | 0 | 0 | Yes |
| *Eurytemora affinis* | 32 | 14 | 10 | 5.7 | 0 | Non-microbial |
| *Neochlamydia* sp. TUME1 | 28 | 13 | 6.2 | 7.5 | 7 | Yes |
| *Bactrocera dorsalis* | 15.4 | 12.1 | 10.9 | 18 | 4.8 | Non-microbial |
| *Glutamicibacter* sp. BW80 | 0 | 0 | 61.1 | 0 | 0 | Yes |
| *Ailuropoda melanoleuca* | 0 | 43.1 | 0 | 17.5 | 0 | Non-microbial |
| *Arthrobacter* sp. PAO19 | 0 | 0 | 60.6 | 0 | 0 | Yes |
| *Arthrobacter* sp. YC-RL1 | 0 | 0 | 58.1 | 0 | 0 | Yes |
| *Psychrobacter* sp. Marseille-P5312 | 0 | 1 | 28.8 | 28.2 | 0 | Yes |
| *Drosophila ananassae* | 13 | 19 | 10 | 11 | 4.7 | Non-microbial |
| *Drosophila navojoa* | 12 | 20 | 5.5 | 11 | 8.9 | Non-microbial |
| *Capra hircus* | 31.7 | 0 | 1 | 23.2 | 1.4 | Non-microbial |
| *Drosophila guanche* | 9.3 | 20.8 | 7.7 | 14.8 | 4.2 | Non-microbial |
| *Pan troglodytes* | 0 | 41.1 | 0 | 15.2 | 0 | Non-microbial |
| *Branchiostoma floridae* | 25.5 | 6.7 | 14.4 | 9.4 | 0 | Non-microbial |
| *Gonium pectorale* | 33 | 8.8 | 8.7 | 5.2 | 0 | Non-microbial |
| *Providencia* sp. WCHPHu000369 | 0 | 50 | 5.4 | 0 | 0 | Yes |
| *Erwinia sp. 198* | 0 | 54.9 | 0 | 0 | 0 | Yes |
| *Boleophthalmus pectinirostris* | 13.9 | 11.7 | 6.8 | 10.7 | 11 | Non-microbial |

| | | | | | | |
|---|---|---|---|---|---|---|
| *Colobus angolensis* | 0 | 35 | 0 | 19 | 0 | Non-microbial |
| *Saitoella complicata* | 0 | 40 | 0 | 13 | 0 | Yes |
| *Saimiri boliviensis* | 0 | 36 | 0 | 17 | 0 | Non-microbial |
| *Bombus terrestris* | 14 | 21 | 2.8 | 9.2 | 5.7 | Non-microbial |
| *Yersiniaceae* bacterium 2015Iso6 | 0 | 52.5 | 0 | 0 | 0 | Yes |
| *Cryptosporidium meleagridis* | 31 | 9 | 6.5 | 4.5 | 0 | Yes |
| *Angomonas deanei* | 16.3 | 12.4 | 6.8 | 15.3 | 0 | Yes |
| *Asbolus verrucosus* | 8.5 | 13 | 5.4 | 17 | 6 | Non-microbial |
| *Epinephelus bruneus* | 0 | 36 | 0 | 13 | 0 | Non-microbial |
| *Mus pahari* | 0 | 39.2 | 0 | 9.3 | 0 | Non-microbial |
| *Psychrobacter* sp. JCM 18902 | 0 | 0 | 18.1 | 30.3 | 0 | Yes |
| *Psychrobacter* sp. C 20.9 | 0 | 0 | 24.6 | 22.8 | 0 | Yes |
| *Pantoea* sp. VS1 | 0 | 21.3 | 0 | 26.1 | 0 | Yes |
| *Curvibacter delicatus* | 0 | 1.4 | 0 | 0 | 46 | Yes |
| *Comamonas thiooxydans* | 18.3 | 11.3 | 3.7 | 13.7 | 0 | Yes |
| *Drosophila pseudoobscura* | 11.6 | 14.5 | 4.1 | 9 | 7.1 | Non-microbial |
| *Drosophila persimilis* | 10 | 12 | 7.4 | 10 | 6.7 | Non-microbial |
| *Shigella flexneri* | 3.8 | 0 | 38.9 | 1.4 | 2 | Yes |
| *Rhodobacter viridis* | 14 | 13 | 3.7 | 12 | 3.2 | Yes |
| *Streptococcus anginosus* | 0 | 0 | 41.3 | 4.1 | 0 | Yes |
| *Leishmania infantum* | 14 | 7.3 | 3 | 20.8 | 0 | Yes |
| *Loxodonta africana* | 0 | 29.5 | 0 | 15.4 | 0 | Non-microbial |
| *Anolis carolinensis* | 9.6 | 21.6 | 6 | 4.5 | 2.8 | Non-microbial |
| *Anopheles gambiae* | 8 | 7.9 | 7.8 | 12 | 8.5 | Non-microbial |
| *Equus caballus* | 0 | 35.6 | 0 | 8.6 | 0 | Non-microbial |
| *Psychrobacter* sp. P11G5 | 0 | 0 | 6.3 | 37.9 | 0 | Yes |
| *Canis lupus* | 0 | 30 | 0 | 14 | 0 | Non-microbial |
| *Rhinopithecus bieti* | 0 | 30 | 0 | 14 | 0 | Non-microbial |
| *Roseateles terrae* | 0 | 8.8 | 0 | 0 | 35 | Yes |
| *Nomascus leucogenys* | 0 | 34 | 0 | 9.3 | 0 | Non-microbial |
| *Enterococcus faecium* | 21.9 | 0 | 0 | 19.6 | 1 | Yes |
| *Pantoea* sp. AG1095 | 0 | 38.5 | 0 | 3.6 | 0 | Yes |
| *Athalia rosae* | 5.9 | 15 | 6.1 | 6.8 | 8.1 | Non-microbial |
| *Acinetobacter johnsonii* | 0 | 41.5 | 0 | 0 | 0 | Yes |
| *Nitrospina gracilis* | 5 | 7.1 | 7.4 | 10 | 12 | Yes |
| *Pantoea septica* | 0 | 40.4 | 0 | 0 | 0 | Yes |
| *Eucalyptus grandis* | 5.8 | 14 | 4.6 | 16 | 0 | Non-microbial |
| *Laodelphax striatellus* | 4.7 | 10 | 4.6 | 14 | 7 | Non-microbial |
| *Drosophila elegans* | 4.2 | 13 | 5.7 | 11 | 6.2 | Non-microbial |
| *Pseudomonas* sp. Ant30-3 | 0 | 0 | 0 | 40 | 0 | Yes |
| *Psychrobacter glacincola* | 0 | 0 | 13.4 | 25.8 | 0 | Yes |
| *Python bivittatus* | 0 | 27 | 0 | 12 | 0 | Non-microbial |
| *Cervus elaphus* | 0 | 27 | 0 | 11 | 0 | Non-microbial |
| *Providencia stuartii* | 0 | 12.2 | 12.3 | 13.4 | 0 | Yes |
| *Pantoea* sp. ARC270 | 0 | 4.9 | 0 | 32.9 | 0 | Yes |

| | | | | | | |
|---|---|---|---|---|---|---|
| *Pantoea* sp. ICBG 1758 | 0 | 21.6 | 0 | 15.7 | 0 | Yes |
| *Mycobacteroides abscessus* | 0 | 9.2 | 20 | 7.8 | 0 | Yes |
| *Mesocricetus auratus* | 0 | 27 | 0 | 10 | 0 | Non-microbial |
| *Pantoea* sp. Sc1 | 0 | 19.4 | 0 | 17.6 | 0 | Yes |
| *Blattella germanica* | 7.5 | 11 | 10 | 4.4 | 4 | Non-microbial |
| *Pantoea* sp. RIT388 | 0 | 36.8 | 0 | 0 | 0 | Yes |
| *Buttiauxella brennerae* | 0 | 0 | 0 | 36.7 | 0 | Yes |
| *Arthrobacter* sp. AG1021 | 0 | 0 | 36.1 | 0 | 0 | Yes |
| *[Curtobacterium] plantarum* | 0 | 26.3 | 0 | 9.8 | 0 | Yes |
| *Pantoea eucrina* | 0 | 36 | 0 | 0 | 0 | Yes |
| *Erwinia* sp. ErVv1 | 2.6 | 23.9 | 3.7 | 5.7 | 0 | Yes |
| *Myotis davidii* | 0 | 28.4 | 0 | 7.5 | 0 | Non-microbial |
| *Myotis lucifugus* | 0 | 27 | 0 | 8.9 | 0 | Non-microbial |
| *Methanosarcina* sp. WWM596 | 8.8 | 9.1 | 1.7 | 9.9 | 6.4 | Yes |
| *Amazona aestiva* | 3.3 | 10.5 | 4.7 | 12.2 | 4.6 | Non-microbial |
| *Acinetobacter indicus* | 0 | 35 | 0 | 0 | 0 | Yes |
| *Avibacterium volantium* | 24 | 0 | 0 | 0 | 10.8 | Yes |
| *Legionella pneumophila* | 7 | 13 | 2.9 | 7.6 | 4.3 | Yes |
| *Psychrobacter* sp. L7 | 0 | 0 | 10.7 | 23.9 | 0 | Yes |
| *Shigella dysenteriae* | 3.5 | 1.5 | 20.5 | 3.9 | 4.3 | Yes |
| *Exiguobacterium sibiricum* | 0 | 0 | 33.6 | 0 | 0 | Yes |
| *Apis cerana* | 9.2 | 6.7 | 3.4 | 8.2 | 5.7 | Non-microbial |
| *Cronobacter sakazakii* | 0 | 20.5 | 0 | 12.6 | 0 | Yes |
| *Wolbachia endosymbiont of Dirofilaria immitis* | 8.5 | 8.1 | 0 | 5.5 | 11 | Yes |
| *Psychrobacter phenylpyruvicus* | 0 | 11 | 15.9 | 5.2 | 0 | Yes |
| *Vombatus ursinus* | 0 | 25.6 | 0 | 6.5 | 0 | Non-microbial |
| *Patagioenas fasciata* | 0 | 23 | 0 | 9.1 | 0 | Non-microbial |
| *Paramormyrops kingsleyae* | 0 | 17.2 | 0 | 14.3 | 0 | Non-microbial |
| *Drosophila takahashii* | 11 | 7.6 | 4.4 | 6.7 | 1.8 | Non-microbial |
| *Trichinella pseudospiralis* | 4.3 | 15 | 0 | 12 | 0 | Yes |
| *Babesia bigemina* | 19.9 | 0 | 1 | 8.1 | 2 | Yes |
| *Acinetobacter* sp. SFA | 0 | 3 | 27.8 | 0 | 0 | Yes |
| *Psychrobacter* sp. Rd 27.2 | 0 | 0 | 9.1 | 21.4 | 0 | Yes |
| *Ooceraea biroi* | 4.6 | 14 | 5.3 | 4.1 | 2.3 | Non-microbial |
| *Chryseobacterium balustinum* | 0 | 0 | 30.2 | 0 | 0 | Yes |
| *Dipodomys ordii* | 0 | 20.8 | 0 | 9.3 | 0 | Non-microbial |
| *Citrobacter braakii* | 2 | 26.1 | 1.8 | 0 | 0 | Yes |
| *Staphylococcus saprophyticus* | 0 | 0 | 26.2 | 3.7 | 0 | Yes |
| *Escherichia albertii* | 2.1 | 0 | 25.9 | 1.9 | 0 | Yes |
| *Psychrobacter* sp. MES7-P7E | 0 | 1.8 | 5.2 | 22.9 | 0 | Yes |
| *Culex quinquefasciatus* | 3.9 | 8.5 | 2.7 | 5.7 | 8.7 | Non-microbial |
| *Wolbachia endosymbiont of Onchocerca ochengi* | 6.1 | 5.5 | 9.4 | 8.2 | 0 | Yes |
| *Psychrobacter fozii* | 0 | 1.3 | 7.5 | 20.2 | 0 | Yes |

| | | | | | | |
|---|---|---|---|---|---|---|
| *Wolbachia endosymbiont of Muscidifurax uniraptor* | 7.2 | 4.4 | 4.6 | 11 | 1.7 | Yes |
| *Enterobacter cancerogenus* | 0 | 22 | 0 | 6.8 | 0 | Yes |
| *Babesia ovata* | 22.7 | 0 | 0 | 4.8 | 1 | Yes |
| *Moraxella atlantae* | 0 | 28.2 | 0 | 0 | 0 | Yes |
| *Type-F symbiont of Plautia stali* | 0 | 26.1 | 0 | 1.9 | 0 | Yes |
| *Psychrobacter* sp. JB193 | 0 | 0 | 13.3 | 14.7 | 0 | Yes |
| *Agrilus planipennis* | 11 | 2.8 | 3.2 | 6.1 | 4.7 | Non-microbial |
| *Leishmania donovani* | 7.5 | 8.1 | 3.1 | 7.2 | 1.4 | Yes |
| *Klebsiella variicola* | 0 | 8 | 0 | 19.1 | 0 | Yes |
| *Novimethylophilus kurashikiensis* | 0 | 0 | 14 | 13 | 0 | Yes |
| *Puma concolor* | 0 | 20 | 0 | 6.9 | 0 | Non-microbial |
| *Lelliottia nimipressuralis* | 0 | 0 | 0 | 26.8 | 0 | Yes |
| *Pantoea* sp. IMH | 0 | 26.7 | 0 | 0 | 0 | Yes |
| *Streptomyces scabiei* | 14 | 3.6 | 2.5 | 6.5 | 0 | Yes |
| *Mandrillus leucophaeus* | 0 | 19 | 0 | 7.5 | 0 | Non-microbial |
| *Eimeria praecox* | 0 | 7.2 | 3.1 | 16 | 0 | Yes |
| *Enterobacter kobei* | 0 | 26.1 | 0 | 0 | 0 | Yes |
| *Rahnella aquatilis* | 0 | 15 | 0 | 10.7 | 0 | Yes |
| *Methylocapsa palsarum* | 0 | 8.9 | 10 | 5.8 | 1 | Yes |
| *Chinchilla lanigera* | 0 | 20 | 0 | 5.6 | 0 | Non-microbial |
| *Yersinia intermedia* | 0 | 25.3 | 0 | 0 | 0 | Yes |
| *Enterobacter asburiae* | 0 | 17.6 | 0 | 7.7 | 0 | Yes |
| *Exiguobacterium* sp. RIT452 | 0 | 0 | 25.1 | 0 | 0 | Yes |
| *Staphylococcus xylosus* | 0 | 0 | 22.2 | 2.8 | 0 | No |
| *Bactrocera tryoni* | 5.3 | 5.1 | 4.1 | 8.4 | 2 | Non-microbial |
| *Arthrobacter* sp. LS16 | 1 | 0 | 23.6 | 0 | 0 | Yes |
| *Agrococcus pavilionensis* | 0 | 0 | 24.5 | 0 | 0 | Yes |
| *Psychrobacter* sp. Cmf 22.2 | 0 | 0 | 6.4 | 18.1 | 0 | Yes |
| *Limulus polyphemus* | 6.4 | 3.8 | 3 | 5.6 | 5.7 | Non-microbial |
| *Pantoea* sp. ICBG 828 | 0 | 0 | 0 | 23.6 | 0 | Yes |
| *Stenotrophomonas maltophilia* | 13.1 | 0 | 10.2 | 0 | 0 | Yes |
| *Yarrowia lipolytica* | 0 | 23.2 | 0 | 0 | 0 | Yes |
| *Pantoea* sp. 111 | 0 | 23 | 0 | 0 | 0 | Yes |
| *Pantoea* sp. PNA 03-3 | 0 | 23 | 0 | 0 | 0 | Yes |
| *Sinorhizobium medicae* | 6.3 | 4.7 | 1 | 11 | 0 | Yes |
| *Clostridium botulinum* | 10.9 | 0 | 0 | 11.9 | 0 | Yes |
| *Chlamydia abortus* | 19.6 | 0 | 0 | 3.1 | 0 | Yes |
| *Macrostomum lignano* | 6.7 | 7.6 | 5 | 3.4 | 0 | Yes |
| *Schistosoma japonicum* | 0 | 13 | 0 | 9.2 | 0 | Yes |
| *Burkholderia mallei* | 8.7 | 3.7 | 1.7 | 7.9 | 0 | Yes |
| *Pteropus vampyrus* | 0 | 16.5 | 0 | 5.4 | 0 | Non-microbial |
| *Corallococcus praedator* | 14 | 3.8 | 4.1 | 0 | 0 | Yes |
| *Kalamiella piersonii* | 0 | 15.4 | 0 | 6.3 | 0 | Yes |
| *Lipoptena fortisetosa* | 2.8 | 6.8 | 3.8 | 3.7 | 4.6 | Non-microbial |

| | | | | | | |
|---|---|---|---|---|---|---|
| *Staphylococcus* sp. OJ82 | 0 | 0 | 17 | 4.6 | 0 | Yes |
| *Serratia fonticola* | 0 | 11 | 0 | 10.6 | 0 | Yes |
| *Psychrobacter pasteurii* | 0 | 0 | 7.1 | 14.4 | 0 | Yes |
| *Buceros rhinoceros* | 0 | 6.8 | 4.7 | 10 | 0 | Non-microbial |
| *Aedes albopictus* | 4.5 | 6.8 | 4.1 | 2.8 | 2.9 | Non-microbial |
| *Arthrobacter* sp. MYb224 | 0 | 0 | 21 | 0 | 0 | Yes |
| *Bacteroides heparinolyticus* | 6.2 | 0 | 7.5 | 1.4 | 5.9 | Yes |
| *Wolbachia endosymbiont of Culex pipiens* | 5.9 | 4.8 | 2.5 | 5.5 | 2.1 | Yes |
| *Propithecus coquereli* | 0 | 19.5 | 0 | 1 | 0 | Non-microbial |
| *Dictyostelium discoideum* | 4.2 | 4.2 | 2.7 | 7.1 | 1.7 | Yes |
| *Escherichia fergusonii* | 1 | 7.5 | 9.1 | 0 | 2 | Yes |
| *Crassostrea gigas* | 0 | 8 | 1.9 | 9.5 | 0 | Non-microbial |
| *Yersinia pseudotuberculosis* | 0 | 19.3 | 0 | 0 | 0 | Yes |
| *Pantoea cypripedii* | 0 | 12.8 | 0 | 6.1 | 0 | Yes |
| *Pantoea* sp. YU22 | 0 | 18.8 | 0 | 0 | 0 | Yes |
| *Curvibacter* sp. GWA2_64_110 | 0 | 0 | 0 | 0 | 18.7 | Yes |
| *Pantoea eucalypti* | 0 | 13.3 | 0 | 5.3 | 0 | Yes |
| *Edwardsiella piscicida* | 2.6 | 7.6 | 2.9 | 4.5 | 1 | Yes |
| *Bicyclus anynana* | 6.3 | 4.8 | 3 | 1.6 | 2.9 | Non-microbial |
| *Tupaia chinensis* | 0 | 13 | 0 | 5.4 | 0 | Non-microbial |
| *Actinobacillus pleuropneumoniae* | 0 | 0 | 0 | 0 | 18.3 | Yes |
| *Rhizophagus* sp. MUCL 43196 | 4 | 7.1 | 3.9 | 3.3 | 0 | Yes |
| *Acinetobacter idrijaensis* | 0 | 18.1 | 0 | 0 | 0 | Yes |
| *Providencia rettgeri* | 0 | 18 | 0 | 0 | 0 | Yes |
| *Hafnia alvei* | 0 | 0 | 0 | 18 | 0 | Yes |
| *Psychrobacter piechaudii* | 0 | 0 | 5.7 | 12 | 0 | Yes |
| *Erwinia tracheiphila* | 0 | 0 | 0 | 17.6 | 0 | Yes |
| *Anaerostipes hadrus* | 6.4 | 0 | 7.4 | 1 | 2.7 | Yes |
| *Psychrobacter* sp. JB385 | 0 | 0 | 5.3 | 12 | 0 | Yes |
| *Neodiprion lecontei* | 2 | 6.1 | 2.8 | 6.4 | 0 | Non-microbial |
| *Aggregatibacter kilianii* | 0 | 0 | 0 | 0 | 17.2 | Yes |
| *Escherichia* sp. MOD1-EC7003 | 0 | 1 | 16.2 | 0 | 0 | Yes |
| *Bacillus subtilis* | 0 | 0 | 4 | 4.9 | 8.2 | Yes |
| *Shigella boydii* | 4.7 | 1 | 7.2 | 3.7 | 0 | Yes |
| *Neisseria meningitidis* | 6.6 | 7.1 | 0 | 2.8 | 0 | Yes |
| *Kluyvera georgiana* | 0 | 6.5 | 0 | 10 | 0 | Yes |
| *Aspergillus ochraceoroseus* | 0 | 5 | 1.9 | 9.4 | 0 | Yes |
| *Drosophila simulans* | 2.7 | 2.7 | 7.8 | 2.9 | 0 | Non-microbial |
| *Harpegnathos saltator* | 4.8 | 6.6 | 2.2 | 2.5 | 0 | Non-microbial |
| *Marinospirillum insulare* | 8 | 5.5 | 2.3 | 0 | 0 | Non-microbial |
| *Acipenser persicus* | 1.8 | 4.5 | 0 | 6.8 | 2.5 | Non-microbial |
| *Corvus cornix* | 12 | 0 | 0 | 3.5 | 0 | Non-microbial |
| *Arthrobacter agilis* | 0 | 0 | 15.5 | 0 | 0 | Yes |
| *Leptomonas seymouri* | 4.6 | 3.8 | 3.7 | 3.3 | 0 | Yes |
| *Methylothermaceae bacteria B42* | 0 | 3.3 | 1.9 | 10.1 | 0 | Yes |

| | | | | | | |
|---|---|---|---|---|---|---|
| *Erwinia billingiae* | 0 | 5.5 | 0 | 9.7 | 0 | Yes |
| *alpha proteobacterium Q-1* | 2 | 6.8 | 6.3 | 0 | 0 | Yes |
| *Haemophilus influenzae* | 0 | 6.2 | 0 | 8.9 | 0 | Yes |
| *Yersiniaceae bacterium 2016Iso3* | 0 | 14.9 | 0 | 0 | 0 | Yes |
| *Trichuris trichiura* | 0 | 0 | 11 | 3.7 | 0 | Yes |
| *Fonticula alba* | 5.8 | 1.5 | 1 | 6.3 | 0 | Yes |
| *Pantoea wallisii* | 0 | 12.7 | 0 | 1.9 | 0 | Yes |
| *Arthrobacter crystallopoietes* | 0 | 0 | 14.5 | 0 | 0 | Yes |
| *Pectobacterium brasiliense* | 0 | 4.8 | 0 | 9.7 | 0 | Yes |
| *Listeria newyorkensis* | 0 | 1 | 7.3 | 6.1 | 0 | Yes |
| *Cimex lectularius* | 3.9 | 3.5 | 0 | 4.4 | 2.5 | Non-microbial |
| *Psychrobacter* sp. 4Bb | 0 | 0 | 0 | 14.3 | 0 | Yes |
| *Elysia chlorotica* | 4.5 | 3.4 | 2 | 1 | 3.3 | Non-microbial |
| *Staphylococcus* sp. AntiMn-1 | 0 | 0 | 14.2 | 0 | 0 | Yes |
| *Avibacterium avium* | 0 | 0 | 0 | 4.3 | 9.8 | Yes |
| *[Eubacterium] rectale* | 1.4 | 6.4 | 4.6 | 1.7 | 0 | Yes |
| *Pantoea coffeiphila* | 0 | 14 | 0 | 0 | 0 | Yes |
| *Pseudomonas nitroreducens* | 14 | 0 | 0 | 0 | 0 | Yes |
| *Erwinia mediterraneensis* | 0 | 14 | 0 | 0 | 0 | Yes |
| *Cronobacter turicensis* | 0 | 14 | 0 | 0 | 0 | Yes |
| *Pantoea allii* | 0 | 1.2 | 0 | 12.6 | 0 | Yes |
| *Camelus bactrianus* | 0 | 8.8 | 0 | 4.9 | 0 | Non-microbial |
| *Oryzias latipes* | 0 | 8.3 | 0 | 5.3 | 0 | Non-microbial |
| *Klebsiella oxytoca* | 0 | 11.1 | 0 | 2.4 | 0 | Yes |
| *Bifidobacterium pseudolongum* | 3.9 | 0 | 6.1 | 2.4 | 1 | Yes |
| *Veillonellaceae* bacterium DNF00626 | 8.5 | 0 | 0 | 4.9 | 0 | Yes |
| *Schistocephalus solidus* | 0 | 2.6 | 1 | 9.7 | 0 | Yes |
| *Leclercia adecarboxylata* | 0 | 4.1 | 0 | 9 | 0 | Yes |
| *Proteus vulgaris* | 0 | 13 | 0 | 0 | 0 | Yes |
| *Paraburkholderia fungorum* | 0 | 13 | 0 | 0 | 0 | Yes |
| *Moraxella lincolnii* | 0 | 13 | 0 | 0 | 0 | Yes |
| *Glaesserella* sp. 15-184 | 0 | 0 | 0 | 0 | 13 | Yes |
| *Staphylococcus succinus* | 0 | 0 | 12.9 | 0 | 0 | Yes |
| *Plasmodium ovale* | 11 | 0 | 0 | 1.9 | 0 | Yes |
| *Exiguobacterium* sp. Leaf196 | 0 | 0 | 12.9 | 0 | 0 | Yes |
| *Staphylococcus* sp. NAM3COL9 | 0 | 0 | 9.9 | 2.9 | 0 | Yes |
| *Gardnerella vaginalis* | 0 | 7 | 5.7 | 0 | 0 | Yes |
| *Lactobacillus sharpeae* | 8.7 | 0 | 2.9 | 1.1 | 0 | Yes |
| *Pantoea* sp. BK028 | 0 | 4.9 | 0 | 7.7 | 0 | Yes |
| *Streptococcus pneumoniae* | 4.9 | 0 | 0 | 7.6 | 0 | Yes |
| *Strigomonas oncopelti* | 0 | 4.2 | 3.9 | 4.3 | 0 | Yes |
| *Arthrobacter oryzae* | 0 | 0 | 12.2 | 0 | 0 | Yes |
| *Enterobacter ludwigii* | 0 | 12.2 | 0 | 0 | 0 | Yes |
| *Staphylococcus capitis* | 0 | 3.3 | 8.8 | 0 | 0 | Yes |
| *Psychrobacter lutiphocae* | 0 | 0 | 8.1 | 4 | 0 | Yes |

| | | | | | | |
|---|---|---|---|---|---|---|
| *Bifidobacterium thermophilum* | 4 | 0 | 4.5 | 1 | 2.6 | Yes |
| *[Erwinia] teleogrylli* | 0 | 12 | 0 | 0 | 0 | Yes |
| *Psychrobacter* sp. AntiMn-1 | 0 | 0 | 3.8 | 8.2 | 0 | Yes |
| *Pluralibacter gergoviae* | 0 | 12 | 0 | 0 | 0 | Yes |
| *Arthrobacter* sp. Hiyo8 | 0 | 0 | 12 | 0 | 0 | Yes |
| *Xanthomonas oryzae* | 11.9 | 0 | 0 | 0 | 0 | Yes |
| *Acinetobacter* sp. RIT592 | 0 | 11.9 | 0 | 0 | 0 | Yes |
| *Psychrobacter* sp. 4Dc | 0 | 0 | 6.3 | 5.5 | 0 | Yes |
| *Chryseobacterium piscium* | 0 | 0 | 11.8 | 0 | 0 | Yes |
| *Wolbachia endosymbiont of Tachinid* sp. | 0 | 3.3 | 0 | 3.3 | 5 | Yes |
| *Pseudarthrobacter phenanthrenivorans* | 0 | 0 | 11.6 | 0 | 0 | Yes |
| *Leishmania major* | 6 | 3.6 | 1.9 | 0 | 0 | Yes |
| *Pantoea stewartii* | 0 | 8.5 | 0 | 2.9 | 0 | Yes |
| *Raoultella planticola* | 0 | 0 | 0 | 11.4 | 0 | Yes |
| *Psychrobacter pygoscelis* | 0 | 6.1 | 0 | 5.3 | 0 | Yes |
| *Cyrtomium guizhouense* | 0 | 4.8 | 0 | 6.5 | 0 | Non-microbial |
| *Rubrivivax* sp. | 6.7 | 4.6 | 0 | 0 | 0 | Yes |
| *Psychrobacter urativorans* | 0 | 5.3 | 0 | 6 | 0 | Yes |
| *Staphylococcus vitulinus* | 0 | 0 | 11.3 | 0 | 0 | Yes |
| *Bifidobacterium longum* | 0 | 0 | 11.3 | 0 | 0 | Yes |
| *Anaplasma phagocytophilum* | 6.1 | 0 | 0 | 5.2 | 0 | Yes |
| *Wolbachia* sp. wMel_KL | 4.4 | 0 | 2.3 | 2.9 | 1.5 | Yes |
| *Superficieibacter electus* | 0 | 9.1 | 2 | 0 | 0 | Yes |
| *Pantoea alhagi* | 0 | 11.1 | 0 | 0 | 0 | Yes |
| *Caballeronia glathei* | 1.4 | 3.6 | 1.5 | 2.9 | 1.6 | Yes |
| *Lactobacillus vaginalis* | 0 | 2 | 5.4 | 3.6 | 0 | Yes |
| *Achromobacter spanius* | 0 | 11 | 0 | 0 | 0 | Yes |
| *Citrobacter* sp. FDAARGOS_156 | 0 | 11 | 0 | 0 | 0 | Yes |
| Escherichia phage L AB-2017 | 0 | 11 | 0 | 0 | 0 | Yes |
| *Erwinia amylovora* | 0 | 0 | 0 | 11 | 0 | Yes |
| *Phaeobacter inhibens* | 0 | 6.9 | 1.3 | 2.7 | 0 | Yes |
| *Cyanobacterium aponinum* | 1.2 | 0 | 3.6 | 1.5 | 4.5 | Non-microbial |
| *Morganella morganii* | 0 | 3.9 | 4.9 | 2 | 0 | Yes |
| *Cardiobacterium hominis* | 0 | 0 | 0 | 10.7 | 0 | Yes |
| *Piliocolobus tephrosceles* | 0 | 9 | 0 | 1.7 | 0 | Non-microbial |
| *Eoetvoesia caeni* | 0 | 1 | 0 | 0 | 9.6 | Yes |
| *Xiphophorus maculatus* | 0 | 2.6 | 1.4 | 6.4 | 0 | Non-microbial |
| *Arthrobacter* sp. MYb227 | 0 | 0 | 10.4 | 0 | 0 | Yes |
| *Plasmodium falciparum* | 0 | 7.1 | 0 | 3.3 | 0 | Yes |
| *Bacteroidetes* bacterium GWC2_40_22 | 5.7 | 3.2 | 0 | 1.4 | 0 | Yes |
| *Psychrobacter alimentarius* | 0 | 0 | 0 | 10.3 | 0 | Yes |
| *Acinetobacter seifertii* | 0 | 2.5 | 2.8 | 4.9 | 0 | Yes |
| *Trichechus manatus* | 0 | 6.5 | 0 | 3.7 | 0 | Non-microbial |
| *Staphylococcus sciuri* | 0 | 0 | 2.6 | 7.4 | 0 | Yes |
| *Bifidobacterium catenulatum* | 0 | 0 | 10 | 0 | 0 | Yes |
| *Rhodanobacter* sp. FW104-R8 | 5.4 | 4.5 | 0 | 0 | 0 | Yes |

| | | | | | | |
|---|---|---|---|---|---|---|
| *Citrobacter portucalensis* | 0 | 9.9 | 0 | 0 | 0 | Yes |
| *Exiguobacterium* sp. KRL4 | 0 | 0 | 9.9 | 0 | 0 | Yes |
| *Pectobacterium punjabense* | 0 | 9.9 | 0 | 0 | 0 | Yes |
| *Galeopterus variegatus* | 0 | 9.8 | 0 | 0 | 0 | Non-microbial |
| *Subdoligranulum* sp. APC924/74 | 1 | 0 | 8.8 | 0 | 0 | Yes |
| *Shewanella* sp. ANA-3 | 0 | 9.8 | 0 | 0 | 0 | Yes |
| *Metarhizium acridum* | 0 | 0 | 0 | 0 | 9.6 | Yes |
| *Psychrobacter* sp. PRwf-1 | 0 | 0 | 0 | 9.5 | 0 | Yes |
| *Aeromonas veronii* | 0 | 9.5 | 0 | 0 | 0 | Yes |
| *Dorea phocaeensis* | 6.4 | 1.4 | 1.7 | 0 | 0 | Yes |
| *Cicer arietinum* | 6.2 | 1.8 | 1.5 | 0 | 0 | Non-microbial |
| *Neomonachus schauinslandi* | 0 | 6.5 | 0 | 3 | 0 | Non-microbial |
| *Izhakiella capsodis* | 0 | 0 | 0 | 9.4 | 0 | Yes |
| *Stenotrophomonas rhizophila* | 9.1 | 0 | 0 | 0 | 0 | Yes |
| *Eufriesea mexicana* | 4.7 | 1 | 3.3 | 0 | 0 | Non-microbial |
| *Pantoea sp. A4* | 0 | 9 | 0 | 0 | 0 | Yes |
| *Mixta gaviniae* | 0 | 0 | 0 | 9 | 0 | Yes |
| Bifidobacterium adolescentis CAG:119 | 2.9 | 0 | 6.1 | 0 | 0 | Yes |
| *Callorhinchus milii* | 0 | 4.9 | 0 | 4 | 0 | Non-microbial |
| *Staphylococcus kloosii* | 0 | 0 | 8.9 | 0 | 0 | Yes |
| *Corvus brachyrhynchos* | 4.4 | 0 | 0 | 4.5 | 0 | Non-microbial |
| *Glutamicibacter soli* | 0 | 0 | 8.9 | 0 | 0 | Yes |
| *Acinetobacter* sp. ACNIH2 | 0 | 8.9 | 0 | 0 | 0 | Yes |
| *Rhodovulum sulfidophilum* | 0 | 3.3 | 3.5 | 2 | 0 | Yes |
| *Felis catus* | 0 | 5.4 | 0 | 3.3 | 0 | Non-microbial |
| *Acinetobacter radioresistens* | 0 | 3 | 5.7 | 0 | 0 | Yes |
| *Pectobacterium fontis* | 0 | 0 | 0 | 8.7 | 0 | Yes |
| *Paeniglutamicibacter antarcticus* | 0 | 0 | 8.7 | 0 | 0 | Yes |
| *Cedecea lapagei* | 0 | 8.5 | 0 | 0 | 0 | Yes |
| *Acinetobacter junii* | 0 | 4.6 | 0 | 3.9 | 0 | Yes |
| *Moraxella boevrei* | 0 | 1 | 0 | 7.5 | 0 | Yes |
| *Drosophila bipectinata* | 0 | 2.8 | 0 | 5.7 | 0 | Non-microbial |
| *Emiliania huxleyi* | 0 | 1.2 | 0 | 7.2 | 0 | Non-microbial |
| *Bacillus sporothermodurans* | 0 | 3.2 | 1.5 | 1 | 2.7 | Yes |
| *Megachile rotundata* | 0 | 2.9 | 1 | 4.4 | 0 | Non-microbial |
| *Kocuria* sp. UCD-OTCP | 0 | 0 | 8.3 | 0 | 0 | Yes |
| *Acinetobacter* sp. CIP 102129 | 0 | 8.2 | 0 | 0 | 0 | Yes |
| *Acinetobacter ursingii* | 0 | 8.2 | 0 | 0 | 0 | Yes |
| *Neisseria cinerea* | 0 | 8.2 | 0 | 0 | 0 | Yes |
| *Leucobacter* sp. OH1287 | 0 | 0 | 8.2 | 0 | 0 | Yes |
| *Rouxiella silvae* | 0 | 8.1 | 0 | 0 | 0 | Yes |
| *Paeniglutamicibacter gangotriensis* | 0 | 0 | 8 | 0 | 0 | Yes |
| *Pseudomonas capeferrum* | 0 | 8 | 0 | 0 | 0 | Yes |
| *Rhodanobacter denitrificans* | 8 | 0 | 0 | 0 | 0 | Yes |
| *Acinetobacter* sp. ANC 5318 | 0 | 8 | 0 | 0 | 0 | Yes |

| | | | | | | |
|---|---|---|---|---|---|---|
| *Curvibacter* sp. PAE-UM | 0 | 0 | 0 | 0 | 8 | Yes |
| *Pedobacter* sp. SM1810 | 0 | 0 | 7.9 | 0 | 0 | Yes |
| *Pantoea* sp. GL120224-02 | 0 | 7.9 | 0 | 0 | 0 | Yes |
| *Haematomicrobium sanguinis* | 0 | 0 | 7.9 | 0 | 0 | Yes |
| *Terrapene carolina* | 0 | 7.8 | 0 | 0 | 0 | Non-microbial |
| *Erwinia iniecta* | 0 | 0 | 0 | 7.8 | 0 | Yes |
| *Enterobacter* sp. HP19 | 0 | 7.8 | 0 | 0 | 0 | Yes |
| *Lactobacillus ruminis* | 1.1 | 0 | 4.7 | 0 | 1.9 | Yes |
| *Candidatus Moranbacteria* bacterium CG23_combo_of_CG06-09_8_20_14_all_39_10 | 0 | 7.7 | 0 | 0 | 0 | Yes |
| *Bacillus cereus* | 0 | 0 | 7.7 | 0 | 0 | Yes |
| *Acinetobacter haemolyticus* | 0 | 7.7 | 0 | 0 | 0 | Yes |
| *Pseudoclavibacter helvolus* | 0 | 0 | 7.7 | 0 | 0 | Yes |
| *Brenneria roseae* | 0 | 0 | 0 | 7.6 | 0 | Yes |
| *Burkholderiales* bacterium RIFCSPHIGHO2_01_FULL_63_240 | 0 | 7.6 | 0 | 0 | 0 | Yes |
| *Pectobacterium parmentieri* | 0 | 7.6 | 0 | 0 | 0 | Yes |
| *Arthrobacter* sp. MYb221 | 0 | 0 | 7.6 | 0 | 0 | Yes |
| *Venturia inaequalis* | 3.2 | 0 | 2.8 | 1.5 | 0 | Yes |
| *Candidimonas* sp. | 0 | 0 | 7.5 | 0 | 0 | Yes |
| *Citricoccus muralis* | 0 | 0 | 7.5 | 0 | 0 | Yes |
| *Corynebacterium efficiens* | 0 | 0 | 7.4 | 0 | 0 | Yes |
| *Enterobacteriaceae* bacterium JKS000234 | 0 | 0 | 0 | 7.4 | 0 | Yes |
| *Bifidobacterium bifidum* | 0 | 0 | 7.4 | 0 | 0 | Yes |
| *Leishmania tarentolae* | 0 | 1.1 | 0 | 6.3 | 0 | Yes |
| *Riemerella anatipestifer* | 0 | 0 | 7.3 | 0 | 0 | Yes |
| Klebsiella cf. planticola B43 | 0 | 7.3 | 0 | 0 | 0 | Yes |
| *Acinetobacter* sp. CIP 101966 | 0 | 7.2 | 0 | 0 | 0 | Yes |
| *Acinetobacter* sp. ANC 4216 | 0 | 7.2 | 0 | 0 | 0 | Yes |
| *Moraxella catarrhalis* | 0 | 7.2 | 0 | 0 | 0 | Yes |
| *Spodoptera litura* | 0 | 2.2 | 0 | 5 | 0 | Non-microbial |
| *Photorhabdus australis* | 0 | 7.2 | 0 | 0 | 0 | Yes |
| *Acinetobacter calcoaceticus* | 0 | 0 | 0 | 7.2 | 0 | Yes |
| *Arthrobacter* sp. RT-1 | 0 | 0 | 7.2 | 0 | 0 | Yes |
| *Pantoea* sp. | 0 | 7.2 | 0 | 0 | 0 | Yes |
| *Trypanosoma equiperdum* | 5.2 | 2 | 0 | 0 | 0 | Yes |
| *Ochotona princeps* | 0 | 5.4 | 0 | 1.7 | 0 | Non-microbial |
| *Pristionchus pacificus* | 4.3 | 2.8 | 0 | 0 | 0 | Yes |
| *Acidovorax citrulli* | 0 | 2 | 0 | 0 | 5.1 | Yes |
| *Streptococcus suis* | 0 | 0 | 0 | 0 | 7.1 | Yes |
| *Pseudomonas kunmingensis* | 7 | 0 | 0 | 0 | 0 | Yes |
| *Parabacteroides merdae* | 0 | 7 | 0 | 0 | 0 | Yes |
| *Acinetobacter* sp. CIP 102136 | 0 | 3.6 | 3.4 | 0 | 0 | Yes |
| *Glutamicibacter* sp. ZJUTW | 0 | 0 | 6.9 | 0 | 0 | Yes |

| | | | | | | |
|---|---|---|---|---|---|---|
| *Tatumella morbirosei* | 0 | 6.9 | 0 | 0 | 0 | Yes |
| *Pantoea* sp. YR343 | 0 | 6.8 | 0 | 0 | 0 | Yes |
| *Arthrobacter* sp. EpRS71 | 0 | 0 | 6.8 | 0 | 0 | Yes |
| *Proteus mirabilis* | 0 | 0 | 0 | 6.8 | 0 | Yes |
| *Moraxella canis* | 0 | 0 | 3.7 | 3 | 0 | Yes |
| *Acinetobacter* sp. MF4642 | 0 | 6.7 | 0 | 0 | 0 | Yes |
| *Psychrobacter* sp. 1501(2011) | 0 | 6.7 | 0 | 0 | 0 | Yes |
| *Serratia odorifera* | 0 | 0 | 0 | 6.7 | 0 | Yes |
| *Paenibacillus popilliae* | 0 | 0 | 6.7 | 0 | 0 | Yes |
| *Arthrobacter glacialis* | 0 | 0 | 6.6 | 0 | 0 | Yes |
| *Enterobacteriaceae* bacterium B14 | 0 | 6.6 | 0 | 0 | 0 | Yes |
| *Escherichia* sp. MOD1-EC7011 | 0 | 0 | 6.5 | 0 | 0 | Yes |
| *Chlamydia psittaci* | 5.5 | 0 | 0 | 1 | 0 | Yes |
| *Shimwellia blattae* | 0 | 6.5 | 0 | 0 | 0 | Non-microbial |
| *Aurantimicrobium* sp. MWH-Mo1 | 0 | 0 | 6.5 | 0 | 0 | Yes |
| *Rothia mucilaginosa* | 0 | 0 | 6.5 | 0 | 0 | Yes |
| *Penaeus vannamei* | 0 | 2 | 0 | 4.4 | 0 | Non-microbial |
| *Psychrobacter pacificensis* | 0 | 0 | 6.4 | 0 | 0 | Yes |
| *Idiomarina aestuarii* | 4.2 | 0 | 0 | 2.2 | 0 | Yes |
| *Varibaculum cambriense* | 0 | 0 | 6.4 | 0 | 0 | Yes |
| *Erwinia gerundensis* | 0 | 0 | 0 | 6.4 | 0 | Yes |
| *Enterobacterales* bacterium CwR94 | 0 | 4.3 | 0 | 2 | 0 | Yes |
| *Corynebacterium pseudopelargi* | 0 | 0 | 6.3 | 0 | 0 | Yes |
| *Pseudomonas kuykendallii* | 0 | 0 | 0 | 2.4 | 3.9 | Yes |
| *Acinetobacter* sp. ANC 4218 | 0 | 6.2 | 0 | 0 | 0 | Yes |
| *Chryseobacterium* sp. 5_R23647 | 0 | 0 | 6.2 | 0 | 0 | Yes |
| *Candidatus* Wolfebacteria bacterium | 3.3 | 2.9 | 0 | 0 | 0 | Yes |
| *Nannospalax galili* | 0 | 4.5 | 0 | 1.6 | 0 | Non-microbial |
| *Cycloclasticus pugetii* | 0 | 0 | 0 | 6.1 | 0 | Yes |
| *Citrobacter* sp. BIDMC107 | 0 | 6.1 | 0 | 0 | 0 | Yes |
| *Chryseobacterium* sp. YR460 | 0 | 0 | 6.1 | 0 | 0 | Yes |
| *Citrobacter koseri* | 0 | 6.1 | 0 | 0 | 0 | Yes |
| *Cyanistes caeruleus* | 0 | 6.1 | 0 | 0 | 0 | Non-microbial |
| *Theropithecus gelada* | 0 | 4.3 | 0 | 1.8 | 0 | Non-microbial |
| *Enterobacter* sp. FY-07 | 0 | 2.2 | 0 | 3.8 | 0 | Yes |
| *Mycobacterium tuberculosis* | 0 | 6 | 0 | 0 | 0 | Yes |
| *Acinetobacter* sp. 883425 | 0 | 6 | 0 | 0 | 0 | Yes |
| *Acinetobacter* sp. WCHA55 | 0 | 6 | 0 | 0 | 0 | Yes |
| *Rhodoferax* sp. OTU1 | 0 | 1 | 0 | 0 | 5 | Yes |
| *Acinetobacter* sp. ACNIH3 | 0 | 6 | 0 | 0 | 0 | Yes |
| *Otolemur garnettii* | 0 | 4.4 | 0 | 1.6 | 0 | Non-microbial |
| *Moraxella caviae* | 0 | 5.9 | 0 | 0 | 0 | Yes |
| *Coprococcus catus* | 0 | 1.8 | 2.8 | 0 | 1.3 | Yes |
| *Staphylococcus* sp. ZWU0021 | 0 | 0 | 2.8 | 3 | 0 | Yes |
| *Cephalophus adersi* | 4.8 | 0 | 0 | 1 | 0 | Non-microbial |

| | | | | | | |
|---|---|---|---|---|---|---|
| *Enterobacter* sp. R1(2018) | 0 | 0 | 0 | 5.8 | 0 | Yes |
| *Sporosarcina* sp. P18a | 0 | 0 | 5.8 | 0 | 0 | Yes |
| *Bemisia tabaci* | 1 | 1.9 | 0 | 1.9 | 1 | Non-microbial |
| *Yersinia rohdei* | 0 | 5.8 | 0 | 0 | 0 | Yes |
| *Staphylococcus felis* | 0 | 0 | 5.8 | 0 | 0 | Yes |
| *Microcystis aeruginosa* | 0 | 0 | 0 | 5.8 | 0 | Non-microbial |
| Escherichia virus P1 | 0 | 5.7 | 0 | 0 | 0 | Yes |
| *Psychrobacter* sp. B29-1 | 0 | 0 | 4.6 | 1.1 | 0 | Yes |
| *Candidatus* Nitrosotalea bavarica | 0 | 5.6 | 0 | 0 | 0 | Yes |
| *Avibacterium gallinarum* | 0 | 0 | 0 | 2.9 | 2.7 | Yes |
| *Paracoccus* sp. N5 | 0 | 0 | 0 | 0 | 5.6 | Yes |
| *Pantoea* sp. AV62 | 0 | 5.6 | 0 | 0 | 0 | Yes |
| *Variovorax* sp. Root473 | 0 | 1.8 | 0 | 0 | 3.8 | Yes |
| *Pseudoalteromonas* sp. TB64 | 0 | 0 | 0 | 5.5 | 0 | Yes |
| *Acinetobacter* sp. AR2-3 | 0 | 5.5 | 0 | 0 | 0 | Yes |
| *Enterococcus hirae* | 5.5 | 0 | 0 | 0 | 0 | Yes |
| *Bifidobacterium merycicum* | 0 | 0 | 4 | 0 | 1.5 | Yes |
| *Paenibacillus* sp. VT-16-81 | 0 | 0 | 5.4 | 0 | 0 | Yes |
| *Vibrio tasmaniensis* | 0 | 0 | 0 | 5.4 | 0 | Yes |
| *Enhydrobacter* sp. 8BJ | 0 | 5.4 | 0 | 0 | 0 | Yes |
| *Yersinia frederiksenii* | 0 | 2 | 0 | 3.4 | 0 | Yes |
| *Carlito syrichta* | 0 | 5.4 | 0 | 0 | 0 | Non-microbial |
| *Acinetobacter* sp. CIP 51.11 | 0 | 5.4 | 0 | 0 | 0 | Yes |
| *Pantoea* sp. OXWO6B1 | 0 | 0 | 0 | 5.4 | 0 | Yes |
| *Arthrobacter* sp. Y81 | 0 | 0 | 5.3 | 0 | 0 | Yes |
| *Brachyspira murdochii* | 5.3 | 0 | 0 | 0 | 0 | Yes |
| *Arthrobacter* sp. ok362 | 0 | 0 | 5.3 | 0 | 0 | Yes |
| *Undibacterium parvum* | 0 | 5.3 | 0 | 0 | 0 | Yes |
| *Fusicatenibacter saccharivorans* | 0 | 0 | 4.3 | 1 | 0 | Yes |
| *Ralstonia insidiosa* | 0 | 5.3 | 0 | 0 | 0 | Yes |
| *Sphingomonas endophytica* | 0 | 0 | 0 | 5.3 | 0 | Yes |
| Staphylococcus virus Sextaec | 0 | 0 | 1.8 | 3.5 | 0 | Yes |
| *Ruegeria* sp. ANG-S4 | 5.2 | 0 | 0 | 0 | 0 | Yes |
| *Agathobaculum butyriciproducens* | 2.7 | 0 | 1.5 | 0 | 1 | Yes |
| *Chryseobacterium* sp. CBo1 | 0 | 0 | 5.2 | 0 | 0 | Yes |
| *Paroedura picta* | 4.2 | 0 | 0 | 1 | 0 | Non-microbial |
| SAR324 cluster bacterium | 0 | 0 | 0 | 5.1 | 0 | Yes |
| *Pseudomonas mendocina* | 0 | 5.1 | 0 | 0 | 0 | Yes |
| *Pantoea rodasii* | 0 | 5.1 | 0 | 0 | 0 | Yes |
| *Xanthomonas citri* | 5.1 | 0 | 0 | 0 | 0 | Yes |
| *Leishmania mexicana* | 5.1 | 0 | 0 | 0 | 0 | Yes |
| Type-D symbiont of Plautia stali | 0 | 0 | 0 | 5 | 0 | Yes |
| *Alligator mississippiensis* | 0 | 3.6 | 0 | 1.4 | 0 | Non-microbial |
| *Mycolicibacterium malmesburyense* | 5 | 0 | 0 | 0 | 0 | Yes |
| *Gallibacterium anatis* | 0 | 5 | 0 | 0 | 0 | Yes |
| *Acinetobacter* sp. MYb10 | 0 | 5 | 0 | 0 | 0 | Yes |

| | | | | | | |
|---|---|---|---|---|---|---|
| *Neisseria lactamica* | 0 | 0 | 0 | 5 | 0 | Yes |
| *Apis dorsata* | 0 | 2 | 0 | 2.9 | 0 | Non-microbial |
| *Acinetobacter* sp. WCHAc060041 | 0 | 4.9 | 0 | 0 | 0 | Yes |
| *Bos taurus* | 1 | 3.9 | 0 | 0 | 0 | Non-microbial |
| *Gordonia polyisoprenivorans* | 0 | 0 | 4.8 | 0 | 0 | Yes |
| *Xenopus tropicalis* | 0 | 4.8 | 0 | 0 | 0 | Non-microbial |
| *Nitrosovibrio* sp. Nv17 | 1 | 0 | 0 | 3.8 | 0 | Yes |
| *Bifidobacterium angulatum* | 1 | 0 | 1.8 | 0 | 2 | Yes |
| *Moraxella* sp. RCAD0137 | 0 | 4.7 | 0 | 0 | 0 | Yes |
| *Acinetobacter schindleri* | 0 | 4.7 | 0 | 0 | 0 | Yes |
| *Meriones unguiculatus* | 0 | 4.7 | 0 | 0 | 0 | Non-microbial |
| *Bacillus lacisalsi* | 0 | 0 | 0 | 4.7 | 0 | Yes |
| *Bifidobacterium* sp. N4G05 | 0 | 0 | 4.7 | 0 | 0 | Yes |
| *Proteus* sp. HMSC14B05 | 0 | 0 | 0 | 4.7 | 0 | Yes |
| *Abrus precatorius* | 0 | 0 | 0 | 4.7 | 0 | Non-microbial |
| *Acinetobacter* sp. ANC 4862 | 0 | 4.7 | 0 | 0 | 0 | Yes |
| *Streptomyces violaceusniger* | 4.6 | 0 | 0 | 0 | 0 | Yes |
| *Macrococcus caseolyticus* | 0 | 0 | 3.6 | 1 | 0 | Yes |
| *Clostridioides difficile* | 1 | 0 | 0 | 3.6 | 0 | Yes |
| *Brachybacterium* sp. UMB0905 | 0 | 0 | 4.6 | 0 | 0 | Yes |
| *Cajanus cajan* | 0 | 4.6 | 0 | 0 | 0 | Non-microbial |
| *Dickeya dadantii* | 0 | 0 | 0 | 4.6 | 0 | Yes |
| *Arthrobacter* sp. yr096 | 0 | 0 | 4.6 | 0 | 0 | Yes |
| *Staphylococcus nepalensis* | 0 | 0 | 4.6 | 0 | 0 | Yes |
| *Roseburia inulinivorans* | 0 | 4.6 | 0 | 0 | 0 | Yes |
| *Escherichia* sp. MOD1-EC5189 | 0 | 0 | 4.6 | 0 | 0 | Yes |
| *Drosophila buzzatii* | 0 | 1 | 0 | 0 | 3.5 | Non-microbial |
| *Acinetobacter tandoii* | 0 | 4.5 | 0 | 0 | 0 | Yes |
| *Pectobacterium aquaticum* | 0 | 0 | 0 | 4.5 | 0 | Yes |
| *Alicycliphilus* sp. B1 | 0 | 0 | 0 | 0 | 4.4 | Yes |
| *Bacillus glycinifermentans* | 0 | 0 | 4.4 | 0 | 0 | Yes |
| *Streptomyces* sp. ZS0098 | 0 | 0 | 4.4 | 0 | 0 | Yes |
| *Bartonella quintana* | 0 | 0 | 0 | 4.4 | 0 | Yes |
| *Kosakonia radicincitans* | 0 | 4.4 | 0 | 0 | 0 | Yes |
| *Acinetobacter pittii* | 0 | 4.4 | 0 | 0 | 0 | Yes |
| *Corynebacterium* sp. HMSC073H12 | 0 | 0 | 4.4 | 0 | 0 | Yes |
| *Exiguobacterium* sp. RIT594 | 0 | 0 | 4.3 | 0 | 0 | Yes |
| *Microbacterium* sp. Leaf159 | 0 | 0 | 4.2 | 0 | 0 | Yes |
| *Tatumella citrea* | 0 | 0 | 0 | 4.2 | 0 | Yes |
| *Collinsella tanakaei* | 0 | 0 | 4.2 | 0 | 0 | Yes |
| *Betaproteobacteria* bacterium *HGW-Betaproteobacteria-18* | 0 | 1.1 | 0 | 0 | 3.1 | Yes |
| *Patescibacteria group* bacterium | 0 | 4.1 | 0 | 0 | 0 | Yes |
| *Moraxella bovis* | 0 | 0 | 4.1 | 0 | 0 | Yes |
| *[Ruminococcus] torques* | 0 | 1 | 2.1 | 1 | 0 | Yes |

| | | | | | | |
|---|---|---|---|---|---|---|
| *Flammeovirga yaeyamensis* | 0 | 2.4 | 1.6 | 0 | 0 | Yes |
| *Acinetobacter soli* | 0 | 4 | 0 | 0 | 0 | Yes |
| *Candidatus Kaiserbacteria* bacterium CG10_big_fil_rev_8_21_14_0_10_49_17 | 2.4 | 1.6 | 0 | 0 | 0 | Yes |
| *Acinetobacter baylyi* | 0 | 4 | 0 | 0 | 0 | Yes |
| *Psychrobacter* sp. JCM 18900 | 0 | 0 | 1 | 3 | 0 | Yes |
| *Pseudarthrobacter sulfonivorans* | 0 | 0 | 0 | 3.9 | 0 | Yes |
| *Marinomonas aquimarina* | 0 | 3.9 | 0 | 0 | 0 | Yes |
| *Acinetobacter wuhouensis* | 0 | 3.9 | 0 | 0 | 0 | Yes |
| *Acinetobacter calcoaceticus/baumannii complex* sp. ABNIH27 | 0 | 3.9 | 0 | 0 | 0 | Yes |
| *Dendroctonus ponderosae* | 0 | 3.9 | 0 | 0 | 0 | Non-microbial |
| *Bartonella tribocorum* | 0 | 0 | 2.9 | 1 | 0 | Yes |
| *Achromobacter pulmonis* | 0 | 1.4 | 0 | 0 | 2.5 | Yes |
| *Brugia malayi* | 3.9 | 0 | 0 | 0 | 0 | Yes |
| *Comamonadaceae* bacterium CG17_big_fil_post_rev_8_21_14_2_50_60_13 | 0 | 1.1 | 0 | 0 | 2.7 | Yes |
| *Klebsiella aerogenes* | 0 | 1 | 2.8 | 0 | 0 | Yes |
| *Arthrobacter enclensis* | 0 | 0 | 3.8 | 0 | 0 | Yes |
| *Acinetobacter* sp. ANC 4281 | 0 | 3.8 | 0 | 0 | 0 | Yes |
| *Yersinia enterocolitica* | 0 | 0 | 0 | 3.7 | 0 | Yes |
| Pseudoalteromonas phage PHS3 | 0 | 0 | 0 | 3.7 | 0 | Yes |
| *Desertihabitans brevis* | 0 | 0 | 3.7 | 0 | 0 | Yes |
| Staphylococcus phage VB-SauS-SA2 | 0 | 0 | 0 | 3.7 | 0 | Yes |
| *Limnohabitans* sp. G3-2 | 0 | 1.4 | 0 | 0 | 2.3 | Yes |
| *Pasteurella multocida* | 0 | 0 | 1.6 | 1 | 1 | Yes |
| *Arcobacter trophiarum* | 0 | 0 | 0 | 3.6 | 0 | Yes |
| *Pseudorhodoferax* sp. Leaf267 | 0 | 0 | 0 | 0 | 3.6 | Yes |
| *Marinobacter pelagius* | 0 | 0 | 3.6 | 0 | 0 | Yes |
| Orf virus | 3.6 | 0 | 0 | 0 | 0 | Yes |
| *Staphylococcus petrasii* | 0 | 0 | 3.6 | 0 | 0 | Yes |
| *Psychrobacter piscatorii* | 0 | 0 | 0 | 3.6 | 0 | Yes |
| *Dysgonamonadaceae bacterium* | 0 | 0 | 3.6 | 0 | 0 | Yes |
| *Toxoplasma gondii* | 1.7 | 0 | 0 | 0 | 1.9 | Yes |
| *Cavia porcellus* | 0 | 3.5 | 0 | 0 | 0 | Non-microbial |
| *Stenotrophomonas indicatrix* | 3.5 | 0 | 0 | 0 | 0 | Yes |
| *Symbiodinium microadriaticum* | 0 | 1.6 | 0 | 1.9 | 0 | Non-microbial |
| *Novosphingobium* sp. | 0 | 3.5 | 0 | 0 | 0 | No |
| *Acinetobacter* sp. ANC 5324 | 0 | 3.4 | 0 | 0 | 0 | Yes |
| *Bifidobacterium animalis* | 0 | 0 | 3.4 | 0 | 0 | Yes |
| *Psychrobacter* sp. TB47 | 0 | 0 | 0 | 3.4 | 0 | Yes |
| *Acinetobacter guillouiae* | 0 | 3.4 | 0 | 0 | 0 | Yes |
| *Trabulsiella odontotermitis* | 0 | 0 | 3.4 | 0 | 0 | Yes |
| *Vibrio splendidus* | 0 | 0 | 0 | 3.4 | 0 | Yes |

| | | | | | | |
|---|---|---|---|---|---|---|
| *Pseudomonadales* bacterium 32-42-5 | 0 | 3.4 | 0 | 0 | 0 | Yes |
| *Paenibacillus macerans* | 0 | 3.4 | 0 | 0 | 0 | Yes |
| *Methyloversatilis* sp. 12-65-5 | 0 | 2 | 0 | 0 | 1.4 | Yes |
| *Acinetobacter* sp. ACNIH4 | 0 | 3.3 | 0 | 0 | 0 | Yes |
| *Erinaceus europaeus* | 0 | 2.3 | 0 | 1 | 0 | Non-microbial |
| *Mycetocola* sp. 622 | 0 | 0 | 3.3 | 0 | 0 | Yes |
| *Eubacterium eligens* | 3.2 | 0 | 0 | 0 | 0 | Yes |
| *Sulfurospirillum arcachonense* | 0 | 3.2 | 0 | 0 | 0 | Yes |
| *Chryseobacterium aquaticum* | 0 | 0 | 3.2 | 0 | 0 | Yes |
| *Serratia* sp. S40 | 0 | 0 | 0 | 3.2 | 0 | Yes |
| *Rhodopirellula sallentina* | 0 | 3.2 | 0 | 0 | 0 | Yes |
| *Haemophilus massiliensis* | 0 | 3.2 | 0 | 0 | 0 | Yes |
| *Acinetobacter* sp. ANC 5347 | 0 | 3.2 | 0 | 0 | 0 | Yes |
| *Psychrobacter* sp. P11G3 | 0 | 0 | 0 | 3.2 | 0 | Yes |
| *Escherichia* sp. MOD1-EC5495 | 0 | 0 | 3.2 | 0 | 0 | Yes |
| *Psychrobacter* sp. Choline-3u-12 | 0 | 0 | 0 | 3.1 | 0 | Yes |
| *Macrococcus goetzii* | 0 | 0 | 3.1 | 0 | 0 | Yes |
| *Acinetobacter* sp. ANC 4204 | 0 | 3.1 | 0 | 0 | 0 | Yes |
| Acinetobacter phage vB_AbaM_B9 | 0 | 0 | 0 | 3.1 | 0 | Yes |
| *Escherichia* sp. MOD1-EC5287 | 0 | 0 | 3.1 | 0 | 0 | Yes |
| *Curvibacter gracilis* | 0 | 0 | 0 | 0 | 3 | Yes |
| *Xanthomonas arboricola* | 3 | 0 | 0 | 0 | 0 | Yes |
| *Albidiferax* sp. OV413 | 0 | 0 | 0 | 0 | 3 | Yes |
| *Ewingella americana* | 0 | 0 | 3 | 0 | 0 | Yes |
| *Actinobacillus seminis* | 0 | 0 | 3 | 0 | 0 | Yes |
| *Enterobacter roggenkampii* | 0 | 0 | 0 | 3 | 0 | Yes |
| *Octodon degus* | 0 | 1.9 | 0 | 1 | 0 | Non-microbial |
| *Xanthomonadales* bacterium CG_4_9_14_3_um_filter_62_6 | 0 | 2.9 | 0 | 0 | 0 | Yes |
| *Exiguobacterium* sp. N4-1P | 0 | 0 | 2.9 | 0 | 0 | Yes |
| *Candidatus Kaiserbacteria* bacterium RIFCSPHIGHO2_02_FULL_56_30 | 1.9 | 1 | 0 | 0 | 0 | Yes |
| *Acinetobacter* sp. CIP 102159 | 0 | 2.9 | 0 | 0 | 0 | Yes |
| *Serratia* sp. S1B | 0 | 2.9 | 0 | 0 | 0 | Yes |
| Psychrobacter phage pOW20-A | 0 | 0 | 0 | 2.8 | 0 | Yes |
| Cloning vector pUC18_LexA+Cp190 | 0 | 0 | 2.8 | 0 | 0 | Yes |
| *Arthrobacter alpinus* | 0 | 0 | 2.8 | 0 | 0 | Yes |
| *Citrobacter amalonaticus* | 0 | 0 | 2.8 | 0 | 0 | Yes |
| *Burkholderia* sp. HI2714 | 2.8 | 0 | 0 | 0 | 0 | Yes |
| *Firmicutes* bacterium CAG:24 | 0 | 0 | 0 | 0 | 2.8 | Yes |
| *Bacillus* sp. LYLB4 | 0 | 0 | 0 | 2.7 | 0 | Yes |
| *Bifidobacterium scaligerum* | 0 | 0 | 0 | 2.7 | 0 | Yes |
| *Burkholderiales* bacterium RIFCSPLOWO2_12_FULL_61_40 | 0 | 0 | 0 | 0 | 2.7 | Yes |

| | | | | | | |
|---|---|---|---|---|---|---|
| *Citrobacter freundii complex* sp. CFNIH2 | 1 | 0 | 1.7 | 0 | 0 | Yes |
| *Polaromonas* sp. AER18D-145 | 0 | 0 | 0 | 0 | 2.7 | Yes |
| *Chryseobacterium scophthalmum* | 0 | 0 | 2.7 | 0 | 0 | Yes |
| *Acinetobacter* sp. ACNIH1 | 0 | 2.5 | 0 | 0 | 0 | Yes |
| *Moraxella equi* | 0 | 0 | 0 | 2.5 | 0 | Yes |
| *Bacillus amyloliquefaciens* | 0 | 0 | 0 | 2.5 | 0 | Yes |
| *Exiguobacterium antarcticum* | 0 | 0 | 2.5 | 0 | 0 | Yes |
| *Verrucomicrobia bacterium* | 0 | 2.5 | 0 | 0 | 0 | Yes |
| *Chryseobacterium taichungense* | 0 | 0 | 2.5 | 0 | 0 | Yes |
| *Bordetella bronchiseptica* | 2.4 | 0 | 0 | 0 | 0 | Yes |
| *Parcubacteria group* bacterium GW2011_GWB1_40_14 | 2.4 | 0 | 0 | 0 | 0 | Yes |
| *Planctomycetes* bacterium RBG_13_62_9 | 0 | 0 | 0 | 0 | 2.4 | Yes |
| *Acinetobacter* sp. VT 511 | 0 | 0 | 2.4 | 0 | 0 | Yes |
| *Blautia obeum* | 0 | 0 | 0 | 0 | 2.4 | Yes |
| *Firmicutes* bacterium AF16-15 | 0 | 0 | 0 | 0 | 2.4 | Yes |
| *Lachnospiraceae* bacterium AM26-1LB | 2.4 | 0 | 0 | 0 | 0 | Yes |
| *Shinella* sp. JR1-6 | 0 | 2.3 | 0 | 0 | 0 | Yes |
| *Eubacterium* sp. CAG76_36_125 | 0 | 0 | 2.3 | 0 | 0 | Yes |
| *Aquabacterium parvum* | 0 | 2.3 | 0 | 0 | 0 | Yes |
| *Rhizobiales bacterium* | 0 | 2.2 | 0 | 0 | 0 | Yes |
| *Avibacterium endocarditidis* | 0 | 0 | 0 | 0 | 2.2 | Yes |
| *Candidatus Nitrosotalea okcheonensis* | 0 | 2.2 | 0 | 0 | 0 | Yes |
| *Chryseobacterium gambrini* | 0 | 0 | 2.2 | 0 | 0 | Yes |
| *Tyzzerella nexilis* | 0 | 0 | 0 | 0 | 2.2 | Yes |
| *Paenibacillus* sp. IHB B 3084 | 0 | 2.2 | 0 | 0 | 0 | Yes |
| *Gracilibacillus dipsosauri* | 0 | 0 | 0 | 2.2 | 0 | Yes |
| *Bifidobacterium pseudocatenulatum* CAG:263 | 0 | 0 | 2.1 | 0 | 0 | Yes |
| *Citrobacter* sp. CFNIH10 | 0 | 0 | 2.1 | 0 | 0 | Yes |
| *Salmonella bongori* | 0 | 0 | 2.1 | 0 | 0 | Yes |
| Phage DP-2017a | 0 | 0 | 2.1 | 0 | 0 | Yes |
| *Desmodus rotundus* | 0 | 2 | 0 | 0 | 0 | Non-microbial |
| *Serratia liquefaciens* | 0 | 2 | 0 | 0 | 0 | Yes |
| *Pseudomonas mandelii* | 0 | 0 | 0 | 2 | 0 | Yes |
| *Bifidobacterium* sp. N5G01 | 0 | 0 | 2 | 0 | 0 | Yes |
| *Odocoileus virginianus* | 2 | 0 | 0 | 0 | 0 | Non-microbial |
| *Flavobacterium aquariorum* | 0 | 0 | 2 | 0 | 0 | Yes |
| *Xanthomonas hortorum* | 2 | 0 | 0 | 0 | 0 | Yes |
| *Drosophila serrata* | 0 | 0 | 0 | 2 | 0 | Non-microbial |
| *Lipotes vexillifer* | 0 | 2 | 0 | 0 | 0 | Non-microbial |
| *Streptococcus equi* | 0 | 0 | 2 | 0 | 0 | Yes |
| *Escherichia* sp. MOD1-EC4550 | 0 | 0 | 2 | 0 | 0 | Yes |
| *Lactococcus lactis* | 0 | 0 | 2 | 0 | 0 | Yes |

| | | | | | | |
|---|---|---|---|---|---|---|
| *Salpingoeca rosetta* | 1 | 1 | 0 | 0 | 0 | Yes |
| *Sanguibacter massiliensis* | 0 | 0 | 2 | 0 | 0 | Yes |
| *Bacillus* sp. JFL15 | 0 | 0 | 0 | 0 | 2 | Yes |
| *Acinetobacter* sp. 51m | 0 | 1.9 | 0 | 0 | 0 | Yes |
| *Dasypus novemcinctus* | 0 | 1.9 | 0 | 0 | 0 | Non-microbial |
| *Smittium megazygosporum* | 0 | 1.9 | 0 | 0 | 0 | Yes |
| *Bacteroides thetaiotaomicron* | 0 | 1.9 | 0 | 0 | 0 | Yes |
| *Gulosibacter* sp. 10 | 0 | 0 | 1.9 | 0 | 0 | Yes |
| *Blochmannia endosymbiont of Camponotus (Colobopsis) obliquus* | 0 | 1.9 | 0 | 0 | 0 | Yes |
| *Citrobacter rodentium* | 0 | 0 | 1.8 | 0 | 0 | Yes |
| *Eimeria brunetti* | 0 | 0 | 0 | 0 | 1.8 | Yes |
| *Haemophilus paraphrohaemolyticus* | 0 | 1.7 | 0 | 0 | 0 | Yes |
| *Variovorax* sp. OV084 | 0 | 0 | 0 | 0 | 1.7 | Yes |
| *Gallibacterium genomosp. 2* | 0 | 0 | 0 | 0 | 1.7 | Yes |
| *Methylibium* sp. CF059 | 0 | 1.7 | 0 | 0 | 0 | Yes |
| *Shinella* sp. HZN7 | 0 | 1.6 | 0 | 0 | 0 | Yes |
| *Zhihengliuella halotolerans* | 0 | 0 | 1.6 | 0 | 0 | Yes |
| *Acinetobacter* sp. 1239920 | 0 | 1.6 | 0 | 0 | 0 | Yes |
| *Echinococcus granulosus* | 0 | 1.6 | 0 | 0 | 0 | Yes |
| *Xanthomonas theicola* | 1.6 | 0 | 0 | 0 | 0 | Yes |
| *Pantoea* sp. PNA 14-12 | 0 | 1.6 | 0 | 0 | 0 | Yes |
| *Chryseobacterium hispalense* | 0 | 0 | 1.5 | 0 | 0 | Yes |
| *Faecalibacterium prausnitzii* | 0 | 1.5 | 0 | 0 | 0 | Yes |
| *Bacillus swezeyi* | 0 | 0 | 0 | 0 | 1.4 | Yes |
| *Staphylococcus edaphicus* | 0 | 0 | 0 | 1.4 | 0 | Yes |
| *Perlucidibaca aquatica* | 0 | 1.4 | 0 | 0 | 0 | Yes |
| Erwinia phage ENT90 | 0 | 0 | 0 | 1.2 | 0 | Yes |
| *Escherichia* sp. R18 | 0 | 0 | 0 | 1 | 0 | Yes |
| *Corynebacterium diphtheriae* | 0 | 0 | 0 | 1 | 0 | Yes |
| *Pseudomonas litoralis* | 0 | 0 | 1 | 0 | 0 | Yes |
| *Citrobacter freundii complex* sp. CFNIH9 | 0 | 0 | 1 | 0 | 0 | Yes |
| *Acidocella aminolytica* | 0 | 1 | 0 | 0 | 0 | Yes |
| *Acinetobacter* sp. HA | 0 | 1 | 0 | 0 | 0 | Yes |
| *Onchocerca flexuosa* | 1 | 0 | 0 | 0 | 0 | Yes |
| *Oreochromis niloticus* | 1 | 0 | 0 | 0 | 0 | Yes |
| *Parafilimonas terrae* | 1 | 0 | 0 | 0 | 0 | Yes |
| *Klebsiella quasipneumoniae* | 0 | 1 | 0 | 0 | 0 | Yes |
| *Calypte anna* | 0 | 0 | 1 | 0 | 0 | Non-microbial |
| *Herbaspirillum* sp. VT-16-41 | 0 | 0 | 0 | 0 | 1 | Yes |
| *Glycine soja* | 0 | 1 | 0 | 0 | 0 | Non-microbial |
| *Acidovorax ebreus* | 0 | 0 | 0 | 0 | 1 | Yes |
| *Haemonchus contortus* | 1 | 0 | 0 | 0 | 0 | Yes |
| *Leishmania amazonensis* | 0 | 1 | 0 | 0 | 0 | Yes |

| | | | | | | |
|---|---|---|---|---|---|---|
| *Corchorus olitorius* | 0 | 1 | 0 | 0 | 0 | Non-microbial |
| *Francisella halioticida* | 0 | 0 | 1 | 0 | 0 | Yes |
| *Enterobacter* sp. R4-368 | 0 | 1 | 0 | 0 | 0 | Yes |
| *Pseudomonas caeni* | 0 | 0 | 0 | 1 | 0 | Yes |
| *Branchiostoma belcheri* | 0 | 1 | 0 | 0 | 0 | Non-microbial |
| *Chryseobacterium culicis* | 0 | 0 | 1 | 0 | 0 | Yes |
| *Marinomonas* sp. | 0 | 0 | 1 | 0 | 0 | Yes |
| *Bos indicus x Bos taurus* | 0 | 0 | 0 | 1 | 0 | Non-microbial |
| *Dickeya zeae* | 0 | 0 | 1 | 0 | 0 | Yes |

AT-1: Urumqi, *M. ovinus*; AT-2: Kuqa City, *M. ovinus*; AT-3: Yecheng County, *M. ovinus*; AT-4: Qira County, *M. ovinus*; AT-5: Qira County, Pupae.

## Additional File 2 (Supplementary Figure)

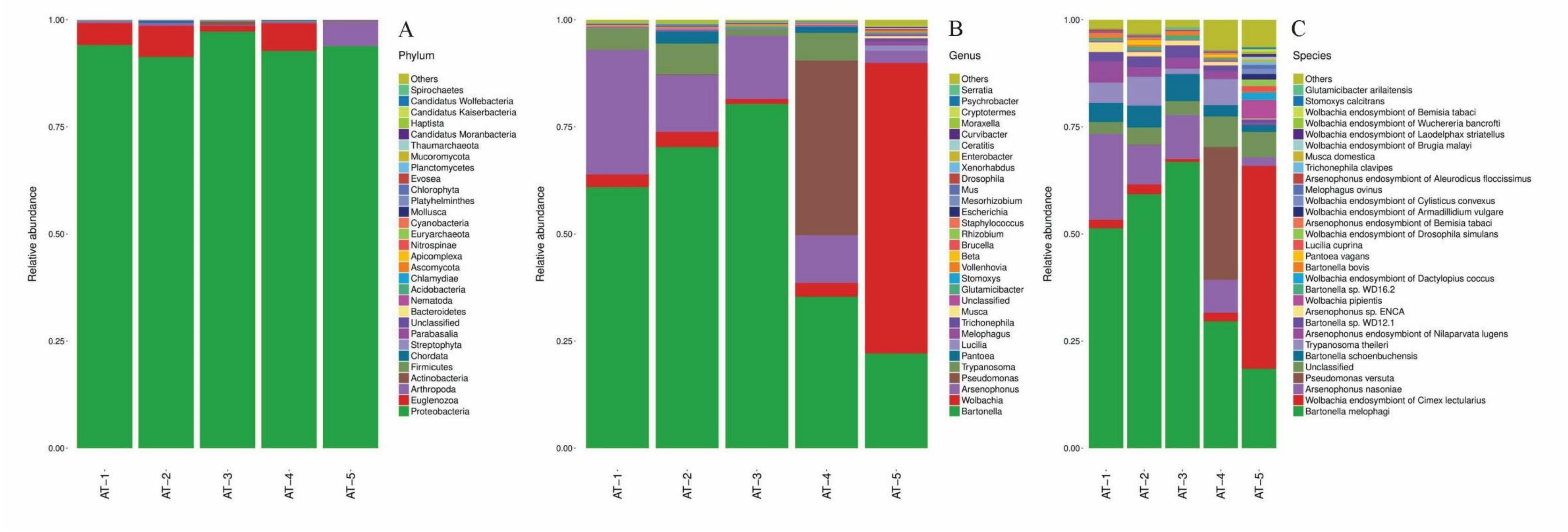


**Figure S1.** A bar plot presenting the microbial community detected in the five samples (**A** Microbial phylum; **B** Microbial Genus; **C** Microbial species).
